# Realization of Robust and Precise Regulation of Gene Expression by Multiple Sigma Recognizable Artificial Promoters

**DOI:** 10.3389/fbioe.2020.00092

**Published:** 2020-02-19

**Authors:** Laichuang Han, Qiaoqing Chen, Qiao Lin, Jintao Cheng, Li Zhou, Zhongmei Liu, Junling Guo, Linpei Zhang, Wenjing Cui, Zhemin Zhou

**Affiliations:** Key Laboratory of Industrial Biotechnology, School of Biotechnology, Jiangnan University, Wuxi, China

**Keywords:** *Bacillus subtilis*, promoter engineering, hybrid promoter, gene expression, sigma factors

## Abstract

Precise regulation of gene expression is fundamental for tailor-made gene circuit design in synthetic biology. Current strategies for this type of development are mainly based on directed evolution beginning with a native promoter template. The performances of engineered promoters are usually limited by the growth phase because only one promoter is recognized by one type of sigma factor (σ). Here, we constructed multiple-σ recognizable artificial hybrid promoters (AHPs) composed of tandems of dual and triple natural minimal promoters (NMPs). These NMPs, which use σ^A^, σ^H^ and σ^W^, had stable functions in different growth phases. The functions of these NMPs resulted from an effect called transcription compensation, in which AHPs sequentially use one type of σ in the corresponding growth phase. The strength of the AHPs was influenced by the combinatorial order of each NMP and the length of the spacers between the NMPs. More importantly, the output of the precise regulation was achieved by equipping AHPs with synthetic ribosome binding sites and by redesigning them for induced systems. This strategy might offer promising applications to rationally design robust synthetic promoters in diverse chassis to spur the construction of more complex gene circuits, which will further the development of synthetic biology.

## Introduction

Synthetic biology accelerates the cross-integration of various disciplines and deepens our understanding of numerous life phenomena in multiple dimensions (Cameron et al., 2014; Church et al., 2014). One of the most important frameworks of synthetic biology is to construct artificial biological systems that can be utilized to produce high-value chemicals in industry and to treat diseases in healthcare (Khalil and Collins, 2010; Keasling, 2012; Breitling and Takano, 2015). To achieve these goals, various biological devices and synthetic circuits have been constructed (Chen et al., 2012). Compared to natural devices and circuits, artificial ones usually exhibited more sensitive and specific. For example, benefit from the lower cross-reactivity and higher sensitivity of engineered parts, independent control of gene expression by 12 inducers could be realized in one cell (Meyer et al., 2019). However, it is still challenging to design reliable devices and circuits because the behaviors of the biological components that regulate the complex circuits are still poorly predictable in the chassis (Zakeri and Carr, 2015; Zhang et al., 2016). Engineered biological components with better performance are expected to decrease the uncertainty of synthetic systems.

Until now, almost all types of components used to regulate gene expression at different levels have been engineered or redesigned, such as promoters, terminators, and transcription factors (TFs) for the regulation of transcription, ribosome binding sites (RBS) and riboswitches for controlling translation and proteolytic tags for protein turnover at the post-translational level (Cameron and Collins, 2014; Mairhofer et al., 2015; Cui et al., 2016; Guiziou et al., 2016; Rossmanith et al., 2018; Castillo-Hair et al., 2019). Among these components, the promoter, as the most fundamental element for transcription initiation, is still the critical genetic element that determines the strength and activation timing of gene expression. To satisfy diverse needs for construction, various types of promoters have been generated and comprehensively evolved for tailor-made functions (Hwang et al., 2018).

Most of the strategies for promoter engineering mainly focus on how to optimize the sequences of single promoters. For instance, the activity of the native promoter is strengthened by substitution of the −35/−10 box for the consensus region, modification of the spacer sequences between the −35/−10 box, and optimization of the sequences of UP elements (Phan et al., 2012; Guan et al., 2016b; Li et al., 2016; Han et al., 2019). Some engineering strategies employ a rational design based on the sequence-function model (Liu et al., 2018) and tandem promoters composed of multiple repeats of core regions (Guan et al., 2016a; Zhang et al., 2017). However, the specific regulation of RNA polymerase (RNAP) by promoters has rarely been considered in these established strategies. Among these subunits of RNAP, sigma factors (σs) specifically bind the core regions of promoters to activate transcription initiation (Lee et al., 2012). Bacteria usually use several kinds of σs to regulate the expression of native genes at different growth phases and in response to diverse milieu (Haldenwang, 1995; Feklistov et al., 2014; Helmann, 2016). These promoters use different σs to mediate non-equivalent levels of transcription along with the growth phases (Pierre et al., 2012), which means that the growth phase-dependent σs limit promoter performance at the phases in which they are inactive. Consequently, engineering of these promoters using single σs is able to increase strength over wide dynamic ranges; however, this type of engineering is unable to enable the promoters to maintain high performance during all growth phases.

According to the SubtiWiki database (Zhu and Stulke, 2018), more than twenty kinds of σs have been identified in *Bacillus subtilis*. These σs perform their functions by synchronizing gene expression to maintain complex physiologies, including vegetative growth, stress response to the milieu, sporulation, competence, and so on (Haldenwang, 1995). σ^A^ is the primary σ and plays an essential role in the expression of the housekeeping genes that keep cells alive, and it serves a similar function to that of σ^70^ in *E. coli* (Haldenwang, 1995). Similar to other σs, σ^B^, and σ^W^ are responsible for responding to intracellular and/or extracellular stress (Hecker et al., 2007; Helmann, 2016). σ^E^, σ^F^, and σ^H^ are involved in tightly regulating sporulation (Patrick and David, 2004). The sigma-54 factor σ^L^ participates in nutrient utilization (Choi and Saier, 2005). Interestingly, although diverse kinds of σs coexist in one cell, they perform biological functions in highly ordered organization (Blom et al., 2011). These precisely regulated factors are promising tools that can be used to exploit orthogonal and robust biological elements. In a recent study, the mutual design of synthetic promoters and the corresponding sigma factors (σs) have been comprehensively established (Bervoets et al., 2018). The σ^B^, σ^F^, and σ^W^ from *B. subtilis* have been heterologously reconstructed in *E. coli* and was used to express genes driven by the σ^B^, σ^F^, and σ^W^-dependent promoters. This toolbox exhibits high orthogonality. Moreover, this toolbox provides a promising principle that engineering the interactions between a promoter and a corresponding σ might confer a more robust function to synthetic promoters.

In this study, we rationally design a framework to develop artificial hybrid promoters (AHPs) with high performance based on natural minimal promoters (NMPs) employing different σs. We designed an array of dual and triple AHPs with combinatorial NMPs using distinct σs. These hybrid promoters activated transcription mediated by the recognition of multiple σs, allowing them to have a higher strength as a result of the compensatory effect between NMPs during different growth phases. More importantly, the output of the precise regulation was achieved by equipping AHPs with a synthetic RBS and by redesigning them as induced systems. This novel strategy might offer promising applications to rationally design robust synthetic promoters in diverse chassis to spur the construction of more complex gene circuits, which will further development of synthetic biology.

## Results and Discussion

### Characterization of Diverse σ-Dependent Natural Minimal Promoters (NMPs)

In general, the expression of a gene is usually driven by a specific promoter recognized by a sole σ factor (Lee et al., 2012). In our previous study, we found that bacterial tandem promoters composed of two or more single promoters displayed higher activity than each of their components. Importantly, tandem promoters comprising different single promoters usually had a higher strength than those composed of the same single promoter (Guan et al., 2016b; Liu et al., 2019). We observed that tandem promoters with higher activity were generally composed of single promoters using distinct σ factors. These appealing observations spurred us to seek to elucidate the general principle for this strategy and to promote its wide utilization to engineer bacterial promoters in a more effective and rational manner.

To this end, natural promoters recognized by different sigma factors were first selected and cloned. Here, to obtain σ-dependent natural promoters with higher activity, we analyzed transcriptomic data to compare the activities of promoters at the transcriptional level. Four groups of genes whose transcription levels depend on the corresponding types of σs (σ^A^, σ^B^, σ^H^, or σ^W^) noted in the database were selected for analysis. As for σ^A^, it regulates genes relevant to vegetative growth and cell division as a primary sigma factor (Haldenwang, 1995). σ^B^ is in charge of handling with general stress (Haldenwang, 1995). σ^H^ is responsible for the expression of genes that regulate sporulation (Haldenwang, 1995). In the stationary phase, cell envelope stress is handled by σ^W^, an extracytoplasmic function (ECF) sigma factor (Helmann, 2016). In total, we analyzed 49, 32, 24, and 23 genes whose transcription levels depend on σ^A^, σ^B^, σ^H^, and σ^W^, respectively, and the datasets are shown in heatmap form (Supplementary Figure S1). In the four datasets, the numbers beside the bars, which correspond to the color, represent promoter activity. The heatmap showed that expression profiles were quite difference between each gene group. Actually, many factors are capable of coooperatively influencing the strength, which are attribute to that outside the sequence-function relationship. Considering that we sought to further construct hybrid promoters using constitutive σ-dependent promoters that required to maximally reduce the complexity of the selected promoters, we ruled out these promoters that potentially be regulated by TFs according to the annotation in DBTBS^1^. Upon this rule, among those σ^A^-dependent transcription genes presented in Supplementary Figure S1a, six promoters from the gene *rpoB*, *sucA*, *mtnK*, *ylbP*, *ylxM*, and *yydE*, which exhibited relatively high strength (indicated by the right brace) were chosen to carry out following studies. The promoters were named after their corresponding gene, P_rpoB_, P_sucA_, P_mtnK_, P_ylbP_, P_ylxM_, and P_yydE_. Likewise, the σ^B^-dependent promoters P_gsiB_, P_relA_, P_yacL_, and P_yqgZ_ (Supplementary Figure S1b) in the σ^B^ dependent group and the σ^H^-dependent promoters P_spoVG_, P_spoVS_, P_spo__0M_ (Supplementary Figure S1c) and P_minC_, as well as the σ^W^-dependent group P_sigW_, P_ydbS_, P_yobJ_, P_yqeZ_, P_ythP_, and P_yuaF_ (Supplementary Figure S1d), were identified to be stronger than others in their respective datasets. Accordingly, a total of 20 promoters that use the four types of σs were further cloned and characterized to authenticate their function.

**FIGURE 1:**
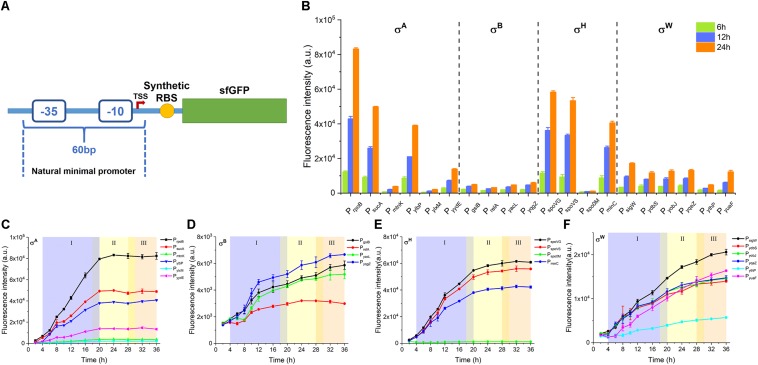
Characterization of candidate NMPs. **(A)** Schematic diagram of NMP cloning and characterization. **(B)** Comparison of sfGFP expression by candidate NMPs at 6, 12, and 24 h after inoculation. **(C)** sfGFP expression by σ^A^ dependent promoters. **(D)** sfGFP expression by σ^B^ dependent promoters. **(E)** sfGFP expression by σ^H^ dependent promoters. **(F)** sfGFP expression by σ^W^ dependent promoters. (I) Exponential growth phase (blue), (II) early stationary phase (yellow), (III) late stationary phase (orange). Error bar means the standard deviation of independent repeats in triplicate.

To rule out the unexpected impact of the upstream element on transcription between different single promoters, we cloned the NMP that was 60 bp in length, including the −35/−10 box and TSS (Figure 1A). All of the sequences and detailed information of the NMPs cloned in this study are listed in Supplementary Table S3. Moreover, all of the candidates were combined with the same synthetic RBS. This design principle ensures an equivalent translation initiation rate for the reporter sfGFP in each expression cassette (Figure 1A). We then measured the strength of all 20 candidates via their fluorescence intensity. Most of the σ^A^-, σ^H^-, and σ^W^-dependent promoters possessed much higher activity than the σ^B^-dependent promoters at 6, 12, and 24 h, except P_mtnk_, P_spo__0M_, and P_ythP_, which had relatively low activity (Figure 1B). P_rpoB_, P_spovG_, and P_sigW_ had the highest activities as σ^A^-, σ^H^-, and σ^W^-dependent promoters, respectively (Figure 1B). According to the expression profiles over the growth processes mediated by the 20 promoters in the four groups, the σ^A^- (Figure 1C) and σ^H^-dependent (Figure 1E) promoters exhibited high activity during the exponential phase (4–20 h). In addition, the σ^H^-dependent promoters performed their functions at a high level during the early stationary phase (18–30 h). Interestingly, the expression mediated by σ^W^-dependent promoters (Figure 1F) was capable of maintaining a continued increase until the late stationary phase (28–36 h). The growth profiles of these recombinant hosts harboring 20 promoters were also determined (Supplementary Figure S2). These data imply that these four types of σs play diverse roles in the regulation of gene expression during different phases. Three promoters, P_rpoB_ (σ^A^-dependent), P_spoVG_ (σ^H^-dependent), and P_sigW_ (σ^W^-dependent), outperformed others, and they were designed to serve as NMPs in the following study.

To verify that these NMPs function through specific binding to their respective σs, rather than binding to more than one σ of the other three sigma factors in the host, we also constructed three σ-deficient (σ^B^, σ^H^, and σ^W^) hosts. The σ^A^-deficient strain was excluded because *B. subtilis* needs σ^A^ to survive (Supplementary Table S1). For σ^B^-dependent promoters, except P_relA_, the activities of P_gsiB_, P_yacL,_ and P_yqgZ_ were obviously suppressed in the σ^B^-deficient stain. Furthermore, σ^H^- and σ^W^-dependent promoters lost almost all activity in the σ^H^- and σ^W^-deficient strains, respectively (Supplementary Figure S3).

### Modular Design and Functional Characterization of AHPs

**FIGURE 2:**
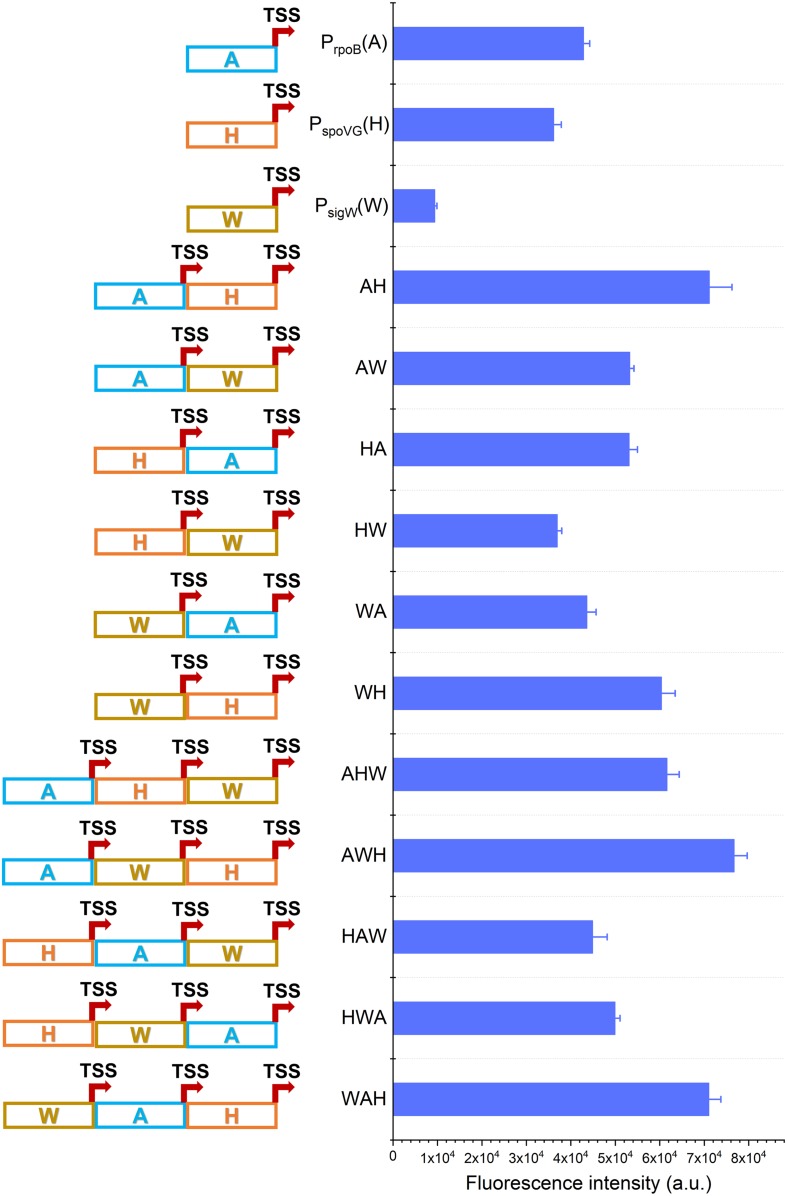
Construction of AHPs. Schematic diagram of AHPs construction and activities characterization of AHPs at12 h. Error bar means the standard deviation of independent repeats in triplicate.

As per the characterization of NMPs presented in Figure 1 and Supplementary Figure S3, we selected three NMPs, P_rpoB_ (σ^A^-dependent, abbreviated as A), P_spoVG_ (σ^H^-dependent, abbreviated as H), and P_sigW_ (σ^W^-dependent, abbreviated as W), that all had high transcription activity for the further construction of AHPs. Here, we constructed the AHPs consist of different σ-dependent NMPs to explore the pattern of regulation by different σs. On the other hand, this design could avoid the potential plasmid deletional instability due to introduction of similar sequences (Hahn and Dubnau, 1985). To demonstrate the universality of the presented engineering strategy, two sets of AHPs, dual and triple promoters, were designed and constructed from NMPs (Figure 2). The same synthetic RBS was used for each module to avoid variations in the translation initiation frequency of sfGFP.

Based on this framework, six dual-version AHPs (AH, AW, HA, HW, WA, and WH) and six triple-version AHPs (AHW, AWH, HAW, HWA, WAH, and WHA) were combinatorially generated. Unfortunately, the plasmid harboring WHA failed to be transformed into *B. subtilis* 168. We speculated that the failure of transformation of a plasmid harboring WHA likely due to the intensive competition of RNAP previously reported on the P_veg_ in *B. subtilis* (Kin, 2011). So WHA was excluded in the following study. The fluorescence intensity revealed that 8 of the 11 AHPs (AH, AW, HA, WH, AHW, AWH, HWA, WAH) were stronger than NMPs during the exponential phase (12 h). The strengths of WA and HAW were equivalent to that of A and higher than W and H, respectively (Figure 2). Interestingly, these AHPs were composed of the same kinds of NMPs, but diverse combinatorial orders exhibited a distinctive strength, implying that the strength of AHPs was also determined by the positions of the NMPs.

### Positional Deployment of NMPs in AHPs Regulates Strength

**FIGURE 3:**
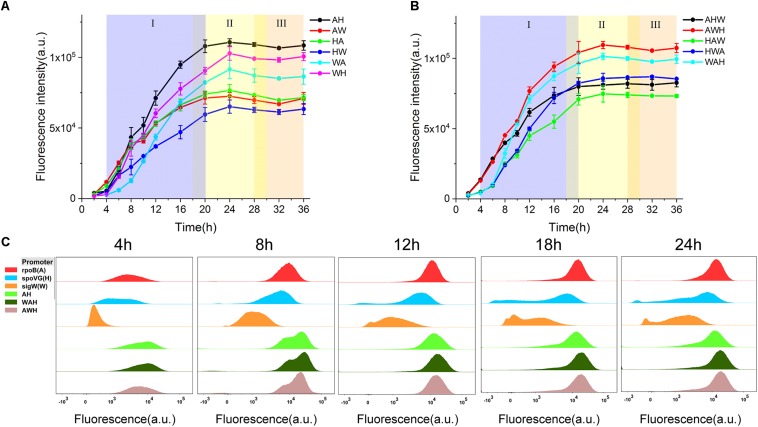
Characterization of AHPs. **(A)** sfGFP expression profiles driven by dual form AHPs. **(B)** sfGFP expression profiles driven by triple form AHPs. **(C)** Three NMPs P_ropB_, P_spoVG_, P_sigW_, and three AHPs AH, WAH, AWH were characterized by FCM. Samples of five timepoints 4, 8, 12, 18, and 24 h cultured at shake flask were harvested and tested. (I) Exponential growth phase (blue), (II) early stationary phase (yellow), (III) late stationary phase (orange). Error bar means the standard deviation of independent repeats in triplicate.

To test the hypothesis that the constituent order of NMPs is a critical determinant of the activity of AHPs, we measured the profiles of cell growth and the expression of sfGFP during culturing mediated by all 11 AHPs and then compared the differential expression profiles among the AHPs (Figures 3A,B and Supplementary Figure S4). According to this rule, the AH, which mediated the highest expression level of sfGFP, was stronger than the NMP-order-reverted version, the HA. This property was maintained over time during the entire culture process. Moreover, the differential expression profiles between AW and WA and WH and HW indicate that the swapping of the position between the two NMPs resulted in a variation of strength (Figure 3A). With regard to the triple version AHPs, when A was fixed upstream of the AHPs, swapping the positions of the H and W resulted in significant expression profile variations. Likewise, a similar regulation occurred between HAW and HWA when the positions of W and A were swapped (Figure 3B). Interestingly, the gene expression profiles of AHPs along with the growth phases were distinct from those of NMPs. For both dual and triple version AHPs, gene expression could be maintained during the early stationary phase (Figures 3A,B), which was evidently better than in the case of σ^A^-dependent promoters (Figure 1C). These results implied that multiple σs participated in the regulation of gene expression during different growth phases. To further study how each NMP influences the performance of AHPs, we selected AH, the strongest dual version AHP, and WAH and AWH, the two strongest triple version AHPs, for further testing.

### AHP Function Relies on the Activation of Each NMP in a Compensatory Manner Regulated by Their Respective σs During Different Growth Phases

The expression levels of sfGFP mediated by the three AHPs (AH, WAH, and AWH) after 4, 8, 12, 18, and 24 h were determined by FCM at the single-cell level. The expression levels of sfGFP mediated by P_rpoB_ (A), P_spoVG_ (H), and P_sigW_ (W) were set as the controls. From the results of the histograms of the fluorescence intensity, we observed that the fluorescence intensity of sfGFP mediated by all three AHPs increased with culture time from 4, 8, 12, 18, and 24 h. Interestingly, the fluorescence intensity results for AH, WAH, and AWH at 8 h displayed two peaks, whereas the fluorescence intensity mediated by the NMPs in the controls did not. This phenomenon indicates that the NMPs recognized by different σs function at different growth phases. In fact, each cell grow under different environment stress even if they were at the same growth phase. Therefore, cell growth will show desynchrony in single cell level, resulting in the differences in gene expression. However, these peaks overlapped after 12 h of culture (Figure 3C). Noticeably, the A module (P_rpoB_), which involves the expression of RNA polymerase subunit β, uses σ^A^ for transcriptional activation during the cell growth process (Boor et al., 1995). This promoter is stronger than those of the H module (P_spoVG_) and W module (P_sigW_) during the exponential phase, whereas this situation was reversed during the stationary phase (Figure 1). Based on these data, we can infer that each NMP module employing phase-associated σ differently contributes to the function of AHPs.

**FIGURE 4:**
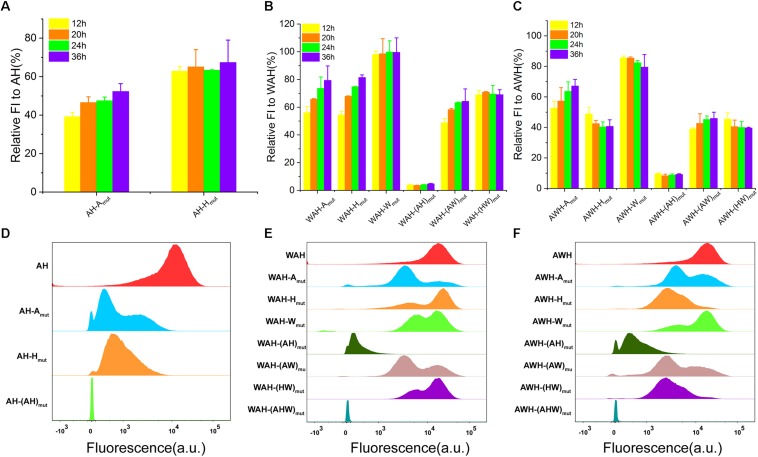
Characterization of AHPs and inactive AHPs. **(A)** Relative FI of partial inactive AH. **(B)** Relative FI of partial inactive WAH. **(C)** Relative FI of partial inactive AWH. **(D–F)** FCM determination of AH, WAH, and AWH and their inactive mutants. Error bar means the standard deviation of independent repeats in triplicate.

To determine the mechanism involving the regulation of AHPs using multiple σs, we designed a series of mutations in AHPs. The genetic context of the synthetic promoter usually influences the properties of transcription (Phan et al., 2012; Rhodius et al., 2012). Therefore, it is important to exclude the effects on characterization. To this end, we designed mutations targeting the NMP module instead of constructing NMP-deletion mutants. These mutants retained a very similar genetic context to the wild-type AHP but lost the function of one module. At first, we searched for suitable mutation sites that can result in a sufficient loss of function of the NMP module. We found that introducing A/G and T/C substitutions for each base-pair of the −10 box led to complete inactivation (Supplementary Figure S5a). Mutations were then applied to AH, WAH, and AWH. We constructed individual NMP mutations and all NMP mutations for the three selected AHPs. As for AH, we constructed AH-A_mut_ (inactivation of A), AH-H_mut_ (inactivation of H), and AH-(AH)_mut_ (inactivation of both A and H, serving as negative control) mutants. Then, a series of AWH and WAH mutants harboring single, dual and triple inactive mutants were also constructed according to this design. The functions of all the mutants were determined by measuring sfGFP expression at the population level and single-cell level. Here, we used the relative FI (the percentage of activity of NMPs mutation promoter to that of whole AHPs) to evaluate the contribution of each NMP to the activity of AHPs. Inactivation of either A or H significantly decreased the strength; however, the extent of the decrease was different. Similarly, mutants both harboring each NMP mutation and dual mutations (AH in Supplementary Figure S5b, WAH in Supplementary Figure S5c and AWH in Supplementary Figure S5d) lost partial activities to different extents compared with the wild-type AHPs. As for the negative controls, AH-(AH)_mut_, WAH-(WAH)_mut_, and AWH-(AWH)_mut_ were completely inactive (Supplementary Figure S5). Furthermore, to verify the function of each NMP at different growth phases, we analyzed the relative FI mediated by partial inactive AHPs at 12, 20, 24, and 36 h (Figure 4). For all AHPs, when A was inactive, the relative FI sustainably increased over the growth. However, when A was the only remaining NMP with activity, the relative FI did not increase after 12 h (Figure 4). These data suggest that the functions of AHPs rely on the performance of each NMP, which contributes to the total strength of AHPs. In addition, mutation of each NMP among the AHP is unable to equal the functioned NMP. That means the function of AH-(A)_mut_ was not completely equivalent to NMP promoter H. These results indicate that the regulation of AHPs was complex instead of simple sum of each NMP. Moreover, according to FCM, these single-NMP mutants derived from AH, as well as the dual-NMP mutants derived from WAH and AWH, exhibited different subpopulations that displayed various fluorescence intensity levels on the histograms (Figures 4D–F). This result implies that the function of AHP relies on the activation of each NMP mediated by the usage of different σs. The limited function of one NMP module in AHPs might be compensated by the rest of the modules using other σ(s).

### Enhancement of the Strength of AHPs by Separating the NMP Modules in AHPs With Suitable Spacer Elements

**FIGURE 5:**
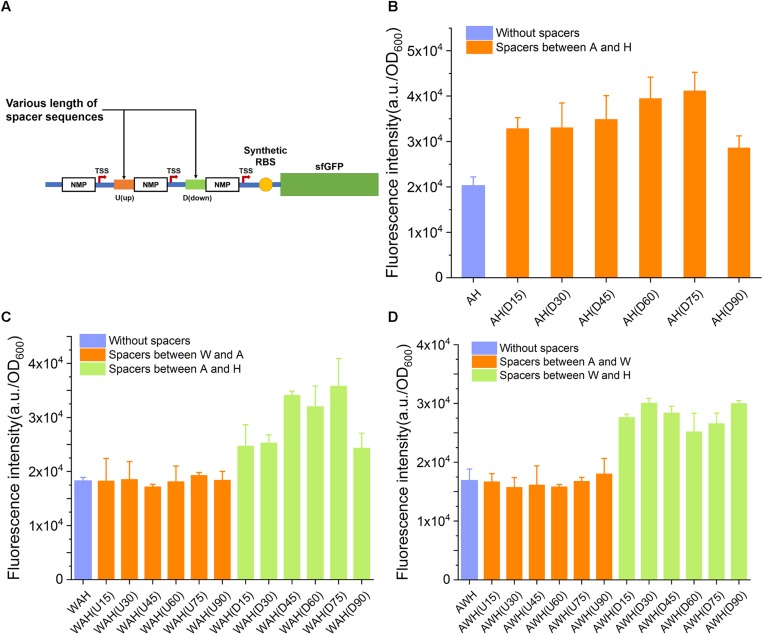
The influence of spacer sequences insertion to the activity of AHPs. **(A)** Schematic diagram of spacer sequences insertion between each NMP of AHP. **(B)** Influence of spacer sequences to dual promoter P_AH_. **(C)** Influence of spacer sequences to triple promoter P_WAH_. **(D)** Influence of spacer sequences to triple promoter P_AWH_. Error bar means the standard deviation of independent repeats in triplicate.

In the aforementioned design, AHPs were constructed with one NMP module next to another, with no space between them. We inferred that the spaced length between NMPs in AHPs might impact the strength of AHPs through altering the internal genetic context. To elucidate this speculation, we first inserted various spacing sequences ranging from 15 to 90 bp in length between each NMP in AH, WAH, and AWH. All of the spacing sequences had a difference of 15 bp in length (for the triple AHPs, when one spacer was varied, another was kept constant) (Figure 5A and Supplementary Table S4). The sequences of these spacers were taken from the native sequences upstream of NMPs on the chromosome. This design generated six variants for AH: two sets of variants for WAH and AWH with a spacer inserted between the W and A and between A and H, respectively. The strength of these modified AHPs was then measured by determining sfGFP expression. The insertion of spacers of the six different lengths increased the strength. As for WAH and AWH, the strength of the promoter was increased by the insertion of spacers between A and H and W and H, respectively (Figures 5B–D). However, for AH(D90) and WAH(D90), spacers longer than 75 bp did not increase the strength, but instead resulted in a lower strength (Figures 5B,C). These results indicate that adding suitable spacer is able to relieve the interference between NMPs and RNAPs. However, longer spacers were unintended for further increasing the activity of AHPs. It is interesting that the spaces between W and A of WAH and A and W of AWH did not make the promoter stronger. A potential reason is that W is a weaker promoter than A and H. Insertion of spacer upstream and downstream of it might contribute slightly to the expression level. In contrast, altering the genetic context between two strong promoters A and H by insertion of suitable spacer resulted in significant increase in expression level. This principle requires more constructs harboring diverse combinatorial strong and weak promoters to reveal the general mechanism. The spacers equipped in AH(75), WAH(D75), and AWH(D30) were considered as the optimal ones. Thus, the three promoters were selected for further evaluation.

### Acquisition of Broad Range and Well-Defined Gene Expression by Combining AHPs With Synthetic RBSs

**FIGURE 6:**
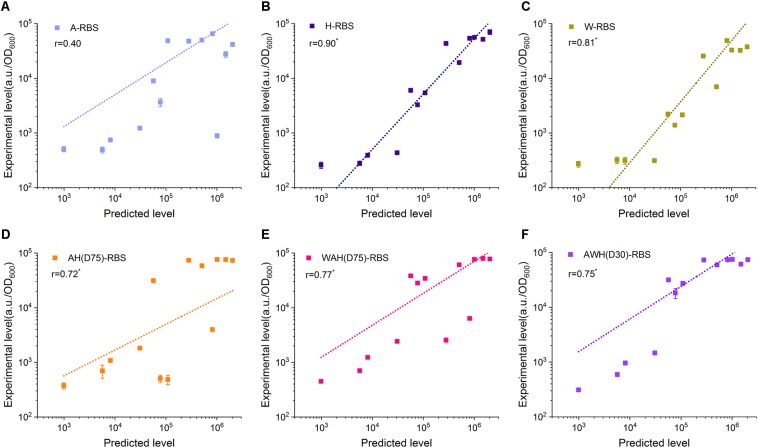
Compatibility between NMPs/AHPs and synthetic RBSs. Thirteen synthetic RBSs were employed to determine the compatibility between A **(A)**, H **(B)**, W **(C)**, AH(D75) **(D)**, WAH(D75) **(E)**, and AWH(D30) **(F)** with synthetic RBSs. The correlations between RBS strength with sfGFP expression were calculated using Pearson’s correlation and the significant difference was analyzed by Student’s *t*-test. * Significant difference at *P* < 0.01 level. Error bar means the standard deviation of independent repeats in triplicate.

Well-defined performance of the biological components determines the function of the newly constructed circuits. Promoter and RBS are two key components in regulation of gene expression at transcriptional level and translational level. However, until now, although several models have been developed to associate the promoter sequence with functional properties, predicting the behavior of arbitrary novel promoters has been challenging for bottom–up design in synthetic biology (Einav and Phillips, 2019; Urtecho et al., 2019). The prediction of the translation initiation rate by the RBS Calculator has been widely used to design suitable RBS sequences to achieve the desired expression level (Mutalik et al., 2013). More importantly, the poor compatibility between promoter and RBS usually render a lot of hurdles in construction of artificial genetic circuits. In many cases, the eventual expression output controlled by a strong promoter combined with a well-defined RBS is still uncertain according to the strength of either the promoter or the RBS (Mutalik et al., 2013). Here, we sought to determine whether the expression level mediated by AHPs can be well-defined by a synthetic RBS. We designed 13 synthetic RBSs with gradient translation initiation rates from a low level (992.72 a.u.) to a high level (2,031,355.3 a.u.) using the RBS Calculator and assembled these synthetic RBSs with P_rpoB_, P_spoVG_, P_sigW_, AH(D75), WAH(D75), and AWH(D30) (Supplementary Figure S5 and Supplementary Table S5). These combinations were divided into six groups according to the promoter. Each group included 13 AHP-RBS pairs. We then measured the fluorescence intensity of sfGFP in each combination. Pearson correlation analysis was employed to evaluate the feature of the expression tendency mediated by the AHPs after equipping them with predicted RBS sequences. The correlation coefficient *r* represents the correlation of the experimentally measured fluorescence intensity to the predicted translation initiation rate (here 0 < |r| < 0.3 means poor correlation, 0.3 < |r| < 0.7 means medium correlation and 0.7 < |r| < 1 means significant correlation). In this case, higher *r* value means the higher correlation between theoretical prediction and experimental results. The P_spoVG_ and P_sigW_ groups had relatively higher *r* values (0.90 and 0.81, respectively) than that of the P_rpoB_ group (0.4) (Figures 6A–C). However, for the AHPs-RBS pairing, AH(D75), WAH(D75), and AWH(D30) had high *r* values (0.72, 0.77, and 0.75) (Figures 6D–F). These results indicate that tuning gene expression level by synthetic RBSs combined with P_rpoB_ is somewhat poorly defined (Figure 6A). For P_spoVG_ and P_sigW_, which had low transcriptional activity, regulation of gene expression using weak RBSs achieved regular tendency (Figures 6B,C). The AHPs possess higher transcriptional activity through well-compatible to the synthetic RBS. it probably benefits from the consistent transcription level of AHPs in different growth phases. The expression output can be precisely defined by designating a desired RBS and then combining it with the AHPs. The results suggest that AHPs are potentially suitable to construct complex genetic circuits that need precisely tune the gene expression level.

### Redesign of AHPs for Different Inducible Systems Reveals the Robust and Modular Properties in a Flexible Genetic Context

**FIGURE 7:**
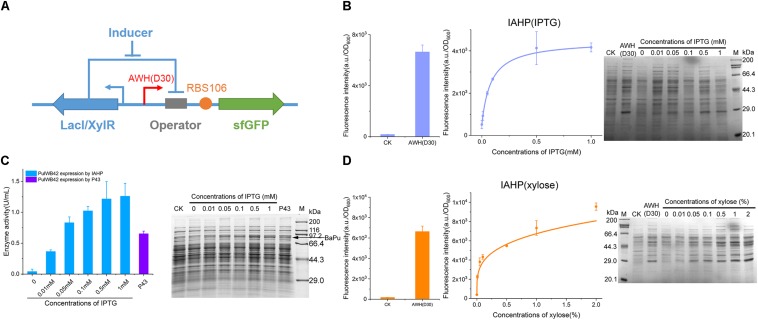
Construction of IAHPs. **(A)** Schematic diagram of the construction of IPTG and xylose inducible AHPs. **(B)** IPTG dose-dependent sfGFP expression by IAHPs. **(C)** Enzyme activity of PulWB42 induced by gradient concentrations of IPTG. After 24 h-induction, cells were harvested by centrifugation and then lysed using lysis buffer and ultrasonic. Enzyme activities were determined by DNS method. PulWB42 expression by strong constitutive promoter P43 was set as comparison. **(D)** Xylose dose-dependent sfGFP expression by IAHPs. The concentration of xylose means the percentage of mass (g) to volume (100 mL). Error bar means the standard deviation of independent repeats in triplicate. CK, plasmid pHT01 without fragments inserted.

For the further development of the repertoire of AHPs, we constructed IPTG- and xylose-induced artificial hybrid promoters (IAHP) employing AWH(D30)-RBS (106) to achieve the desired strength. As shown in Figure 7A, a single copy of the repressor was expressed under the control of the constitutive LacI promoter expressed by the pHT01 plasmid. The corresponding lacO or xylO operator was inserted between AWH(D30) and RBS106 (Figure 7A), generating two inducible systems that responded to IPTG and xylose (the sequences were listed in Supplementary Table S6), respectively. To determine the response of the inducible system to the concentration of the inducer IPTG, we activated sfGFP expression by adding IPTG from 0.01 to 1 mM and set the pHT01 plasmid as the negative control and the constitutive AWH(D30) as the positive control (Figure 7B). The fluorescence intensity (a.u./OD_600_) of each treatment was measured after 24 h of growth/induction. sfGFP was strictly repressed by LacI without IPTG; then, sfGFP expression was enhanced according to the increasing concentration of IPTG (EC_50_ = 0.09 mM), and high concentrations of IPTG activated sfGFP expression at high levels, approaching that of the constitutive AWH(D30), which confirmed that this inducible system was well-regulated by the inducer IPTG (Figure 7B). This result was also verified by SDS-PAGE (Figure 7B). Similarly, in the xylose-induced system, sfGFP expression was able to be tightly tuned by various concentrations of xylose ranging from 0.01 to 2% (across 200-fold), and the EC_50_ was 0.4% (Figure 7D). To identify the versatility of the synthetic element, we substituted sfGFP for another protein, pullulanase. Pullulanase from *Anoxybacillus* sp. WB42 (PulWB42) was inserted into the IPTG-induced IAHP system, and the expression of PulWB42 was successfully activated by various concentrations of IPTG (Figure 7C). Importantly, the expression level of PulWB42 under the control of IAHP was 1.9-fold higher than that using a commonly employed strong constitutive promoter, P43, in *B. subtilis* (Figure 7C). These results indicate that AWH(D30) is broadly compatible with diverse regulatory elements and can be flexibly applied to construct new circuits.

### Rewiring the Synthetic Quorum-Sensing Circuit in *B. subtilis* Employing AHPs

**FIGURE 8:**
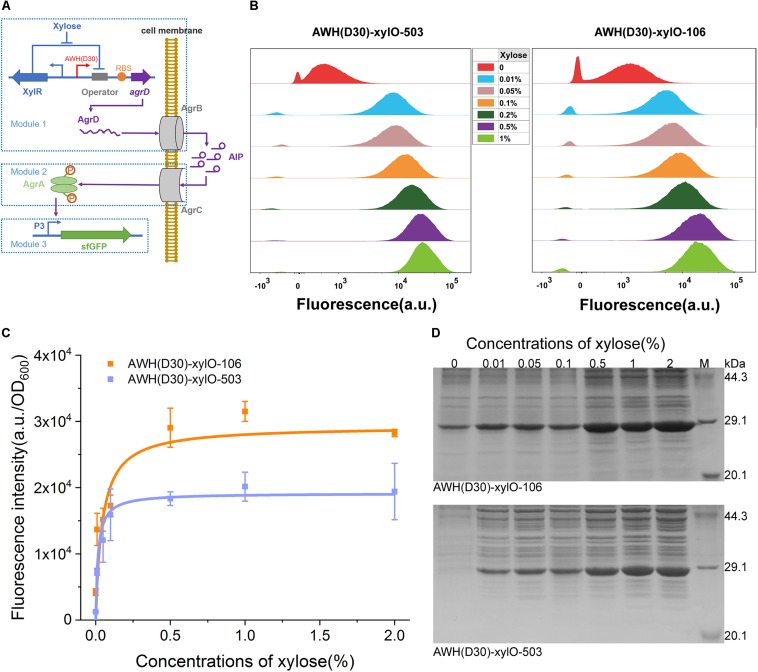
Tuning heterogeneous quorum sensing system in *B. subtilis* using IAHP. **(A)** Schematic diagram of the heterogeneous quorum sensing system controlled by IAHP. **(B)** Characterization of IAHP mediated heterogeneous quorum sensing system using FCM at various concentrations of xylose. **(C)** sfGFP expression profiles by AWH(D30)-xylO-RBS106 and AWH(D30)-xylO-RBS503. **(D)** SDS-PAGE analysis of sfGFP expression. The concentration of xylose means the percentage of mass (g) to volume (100 mL). Error bar means the standard deviation of independent repeats in triplicate.

To further estimate the robustness and versatility of AWH(D30) in the complex circuit, we employed the xylose-inducible IAHP to rewire a quorum sensing (QS), two-component signal transduction system, agr, from *Staphylococcus aureus*. In this system, the auto-inducing peptide (AIP), a cyclic peptide containing an internal thioester bond, was used as the signal molecule (Whitehead et al., 2001; Waters and Bassler, 2005; Novick and Geisinger, 2008). Because the QS signals dynamically activate or suppress gene expression along with cell growth, reconstitution of naturally occurring systems has been used to autonomously induce the expression of enzymes of the biosynthesis pathway in metabolic engineering (Gupta et al., 2017; Doong et al., 2018). The agr QS has previously been shown to be able to function in *Bacillus megatherium* (Marchand and Collins, 2013, 2016). Here, we rewired the agr QS system in *B. subtilis* 168 by redesigning the natural system to include three separate modules. The signal generating module (Module 1), which harbors *agrD*, encoding the precursor of AIP-I, was expressed under the control of xylose-induced AWH(D30)-xylO, and the product AgrD was processed by AgrB to produce AIP-I (Figure 8A). The signal transmission module (Module 2) controlled the sensing of the concentration of AIP-I and activation of the transcriptional factor AgrA through a phosphorylation cascade. The output module (Module 3) expressed sfGFP and was controlled by P3 activated by the phosphorylated AgrA (Figure 8A). To verify the robustness of the synthetic agr QS system whose activation was mediated by AWH(D30)-xylO, two versions of synthetic RBSs with defined predicted levels were paired with AWH(D30)-xylO to express AgrD, yielding two sets of expression elements, AWH(D30)-xylO-503 and AWH(D30)-xylO-106.

The activated profiles of the synthetic agrQS systems were evaluated by adding different concentrations of xylose. FCM was used to measure the level of activation at the single-cell level. Figure 8B shows that the two synthetic agrQS systems were strictly activated by xylose spanning a wide range of concentrations from 0.01 to 1%. At a relatively low xylose concentration (0.01%), both of the systems were dramatically activated at high levels. Increasing the concentration levels of xylose (0.05, 0.1, 0.2, 0.5, and 1%) regularly elevated the activation levels in the systems triggered by both AWH(D30)-xylO-503 and AWH(D30)-xylO-106 (Figure 8B). To further evaluate the dose-dependent manner of the arg QS system, we plotted the activated kinetics of the two systems according to the fluorescence intensity against the concentration via non-linear curves based on Michaelis-Menten kinetics. The two systems had similar activated kinetics profiles for concentrations of xylose from 0.01 to 1%; however, they differed with respect to *K*_m_ and V_max_. The fitted *K*_m_ and V_max_ for AWH(D30)-xylO-503 were 0.05 and 2,334%, respectively, and 0.02 and 3,109% for AWH(D30)-xylO-106 (Figure 8C), indicating that activation of the agrQS by xylose is highly efficient and well-defined. Finally, the expression levels of sfGFP triggered by the systems were further tested by SDS-PAGE, and thus, the output effectiveness was authenticated (Figure 8D).

## Conclusion

We developed a novel strategy for constructing AHPs using NMPs recognized by different σs in the model microorganism *B. subtilis*. The AHPs mediated stronger gene expression throughout cell growth through a transcription compensatory property based on multiple-σ recognition. More importantly, designing AHPs using NMPs with different sequences avoids the potential instability in the construction of plasmids caused by highly efficient homologous recombination. In addition, short NMPs ensure the portability of AHPs, which is crucial for the construction of complex genetic circuits. The AHPs had better compatibility with other regulators and were suitable to construct tunable gene expression systems through combining AHPs with synthetic RBSs and repressors. The output of the precise regulation was achieved by equipping AHPs with synthetic RBSs and by redesigning them for induced systems.

## Materials and Methods

### Strains, Plasmids, and Culture Media

All of the strains and plasmids used in this study are listed in Supplementary Table S1. pBSG03, a shuttle plasmid harboring the reporter GFP driven by promoter P_srfA_, was used as the backbone for screening and characterizing NMPs and AHPs. The shuttle plasmid pHT01 was used as the backbone for the construction of IAHP systems. *E. coli* JM109 was used as the host for plasmid propagation. The wild-type strain *B. subtilis* 168 was used as the host for the determination of activities of promoters, and all recombinant strains were derived from *B. subtilis* 168. The recombinant strain BsBCA, constructed by the integration of the agr quorum sensing genes *agrB*, *agrC*, and *agrA* from *S. aureus* into the chromosome of *B. subtilis* 168, was used to verify the performance of IAHPs in complex synthetic circuits. Luria-Bertani (LB) medium (10 g/L tryptone, 5 g/L yeast extract, 10 g/L NaCl, pH 7.0) was used to culture the hosts. When preparing solid media, 1.5% agar was used. The concentrations of the antibiotics for the selection and growth were added to the media as follows: 100 μg/mL ampicillin; 5 μg/mL kanamycin; 10 μg/mL chloramphenicol; 20 μg/mL zeocin; and 1 μg/mL erythromycin.

### Transcriptome Analysis and Relevant Database

The transcriptome data used in this study to analyze the transcriptional levels of sigma dependent NMPs were obtained from the Gene Expression Omnibus (GEO) of the National Center for Biotechnology Information Search database (NCBI) (GEO accession: GSE19831) (Blom et al., 2011). The transcription levels of selected genes were analyzed and visualized with heatmaps using HemI software (Deng et al., 2014). Sequence information including −35/−10 box and TSS of promoters was obtained from the DBTBS database^2^.

### Construction of NMP-Mediated sfGFP Expression Plasmids

In this study, all plasmids were constructed by using polymerase chain reaction (PCR) and Gibson Assembly as described previously (Han et al., 2019). The primers used in this study are listed in Supplementary Table S2. Homologous arms (20–30 bp) were designed into each primer for seamless ligation using Gibson Assembly.

To construct NMP-mediated sfGFP expression plasmids, the target sequences of the NMPs listed in Supplementary Table S3 were designed into the corresponding primers listed in Supplementary Table S2, and the pBRBS504-sfGFP plasmid harboring the RBS504-sfGFP fragment was employed as the template. The sequences of NMPs were inserted upstream of the RBS504-sfGFP fragment by PCR, and after elimination of the template by *Dpn*I and seamless self-cyclization by Gibson Assembly, the products were transformed into *E. coli* JM109. At last, the recombinant plasmids were further verified by DNA sequencing.

### Construction of σ Deficient Strains

In this study, we constructed σ^B^-, σ^H^-, and σ^W^-deficient strains by Cre-*lox* homologous recombination. At first, ∼600 bp sequences flanking the target genes, which served as homologous left and right arms, were amplified and then cloned into the p7Z6 vector upstream and downstream of the *bleR* gene. The fragments composed of the homologous arms and *bleR* gene were then amplified by PCR and transformed into *B. subtilis* 168. The transformed cells were plated onto LB agar plates with zeocin. Transformants were screened using colony PCR and sequencing to verify that the target genes were knocked out.

### Design and Construction of Artificial Hybrid Promoters (AHPs)

Similar to the clones of NMPs, plasmids harboring 6 dual and 6 triple AHPs were also constructed by PCR and Gibson Assembly mediated self-cyclization.

For the construction of dual AHPs, the plasmids harboring NMPs (Supplementary Table S1) were employed as templates, and another NMP was introduced into the position upstream of the inherent NMP by the primers including the target sequences (Supplementary Table S2). For construction of triple AHPs, the plasmids harboring dual AHPs (Supplementary Table S1) were employed as templates, and the third NMP was introduced into the region upstream of the dual AHPs in the template by the primers including the target sequences (Supplementary Table S2). All of the plasmids harboring AHPs were listed in Supplementary Table S1.

In this study, we inserted an array of spacers of 15 to 90 bp in length (with an increase of 15 bp between each of them) between NMPs to strengthen the activities of AH, WAH, and AWH. The sequence of each spacer was obtained from the chromosomal sequence upstream of each NMP. The fragments of the spacers were genetically fused to primers ranging from 15 to 90 bp with increases of 15 bp in length. All of the spacer sequences are listed in Supplementary Table S4.

### Design and Modular Assembly of Synthetic RBSs of Various Strength to NMPs and AHPs

The synthetic RBSs used in this study were designed by the RBS Calculator^3^ (Salis, 2011) (Supplementary Table S5). The sequences and predicted translation initiation rates of synthetic RBSs are listed in Supplementary Table S5. RBS504, with medium strength, was employed to compare the strengths of NMPs and AHPs. For modular assembly of synthetic RBSs with various strength to NMPs and AHPs, RBS504 was replaced by primers including sequences of other synthetic RBSs (Supplementary Table S2) through PCR and self-cyclization by Gibson Assembly.

### Construction of Inducible Gene Expression Systems Using AHP

In this study, a spacer optimized AHP AWH(D30) was used and combined with the repressors LacI and XylR and their corresponding operators to construct inducible AHPs (Supplementary Table S6). The synthetic RBS with high strength RBS106 was used to ensure gene expression at a high level (Supplementary Table S5). To construct the IPTG inducible system, the P_grac_ of pHT01 was substituted with AWH(D30), and the RBS106-sfGFP fragment was cloned in downstream of *lacO*. For construction of xylose inducible systems, the *lacI* gene was substituted with the *xylR* gene cloned from the pAX01 plasmid, and the corresponding *xylO* was cloned from promoter P_xylA_ to replace *lacO*. The constitutive version without any repressors or operators was also constructed for comparison. The measured fluorescence intensity values (a.u./OD_600_) as a function of inducer concentration were fit to the agonist vs. response equation. EC_50_ was defined as the concentration of inducer required to activate the half-maximal expression of sfGFP.

### Reconstitution of agr Quorum Sensing System Using Xylose-Dependent IAHP in *B. subtilis*

In this study, we constructed the agrQS system composed of three modules (signal generating module, signal transmission module, and output module) into *B. subtilis* 168 (Figure 8A). The signal transmission module including P43-*agrB*-*agrC*-*agrA* cassette was constructed into the chromosome of *B. subtilis* 168. To construct the P43-*agrB*-*agrC*-*agrA* cassette, the constitutive P43 promoter (amplified from the genome of *B. subtilis* 168), *agrB* (amplified from pT7-agrBD-I) and *agrC*-*agrA* cassette (amplified from pXylA-agrCA-I) were firstly cloned into the pAX01 integration vector, yielding pAX-P43-agrBCA. The P43-*agrB*-*agrC*-*agrA* fragment was then amplified by PCR using PlacA-1/PlacA-2 primers and transformed into *B. subtilis* 168. The transformed cells were pooled onto the LB agar plates with erythromycin. At last, the recombinant strain, integrated with P43-*agrB*-*agrC*-*agrA* in the *lacA* site by double crossover, was verified by PCR. Both the signal generating module of the *agrD* gene regulated by the xylose induced system and the output module of sfGFP driven by promoter P3 were constructed into the same plasmid with the opposite expression cassette orientation.

### Assays for Determination of Fluorescence Intensity of sfGFP

The super fold green fluorescent protein (sfGFP) served as the reporter to determine the activities of NMPs, AHPs, and IAHPs (Supplementary Table S6). To determine the activities of the promoters, a two-stage culture method was carried out. At first, a single clone of each recombinant strain was cultured overnight at 37°C with shaking at 200 rpm in test tubes containing 5 mL LB medium. Then, 1 mL of seed liquid was transferred into 250-mL shake flasks containing 50 mL of LB medium, after which the cultures were incubated at 37°C with shaking at 200 rpm. When needed, cells were harvested in appropriate volumes by centrifugation at 8,000 rpm for 5 min to monitor growth and expression of sfGFP. The pellets were then washed three times with phosphate buffered saline (PBS, 8 g/L NaCl, 0.2 g/L KCl, 1.44 g/L Na_2_HPO_4_, 0.24 g/L KH_2_PO_4_, pH 7.4) and resuspended in PBS with appropriate dilution. Each sample (200 μL) was transferred into 96-well black-walled plates and analyzed by a PerkinElmer EnSpire^®^ 2300 Multimode Plate Reader (excitation at 485 nm and emission at 528 nm).

Flow cytometry was employed to test the expression level of sfGFP at the single-cell level. For this approach, *B. subtilis* cells were harvested by centrifugation at 12,000 rpm for 2 min. The pellets were then washed three times by PBS and resuspended in PBS, and the OD_600_ was adjusted to 0.15. Samples were tested using BD FACSAria^TM^ III (Becton, Dickinson and Company, United States). Each cytometric measurement was performed on 100,000 cells, and forward scatter (FSC) was used for trigger. Cell populations were gated according to their FSC and side scatter (SSC) distributions (Supplementary Figure S6).

### Expression and Characterization of PulWB42 Produced by IAHP

The pullulanase gene from *Anoxybacillus* sp. WB42 (PulWB42) was cloned into the IPTG inducible expression plasmid pHT-PAWH-lac-sfGFP by replacing the sfGFP gene. The recombinant strain for production of PulWB42 was inoculated and cultured in 250-mL shake flasks containing 50 mL LB medium with chloramphenicol. PulWB42 was expressed by adding various concentrations of IPTG at 37°C and 200 rpm. The enzymatic activity of PulWB42 was determined by the 3,5-dinitrosalicylic acid (DNS) method as described previously (Wang et al., 2017).

## Data Availability Statement

The authors declare that all the data supporting the findings of this study are available within the paper and its Supplementary Information files or are available from the corresponding author on request.

## Author Contributions

LH and WC conceived the project and designed the experiments. QC, QL, and JC performed the molecular cloning experiments. LZ, ZL, and JG measured the cell growth and GFP expression. LPZ performed FCM and analyzed the results. LH, WC, and ZZ analyzed experiment data and wrote this manuscript.

## Conflict of Interest

The authors declare that the research was conducted in the absence of any commercial or financial relationships that could be construed as a potential conflict of interest.

## Footnotes

1 http://dbtbs.hgc.jp/

2 http://dbtbs.hgc.jp/

3 https://salislab.net/software/
