# Supplementary material for: Realization of Robust and Precise Regulation of Gene Expression by Multiple Sigma Recognizable Artificial Promoters
Source: Front Bioeng Biotechnol. 2020 Feb 19;8:92. doi: 10.3389/fbioe.2020.00092 (PMC7042180; doi:10.3389/fbioe.2020.00092)
Supplement: Supplementary file 1 [file Data_Sheet_1.docx]

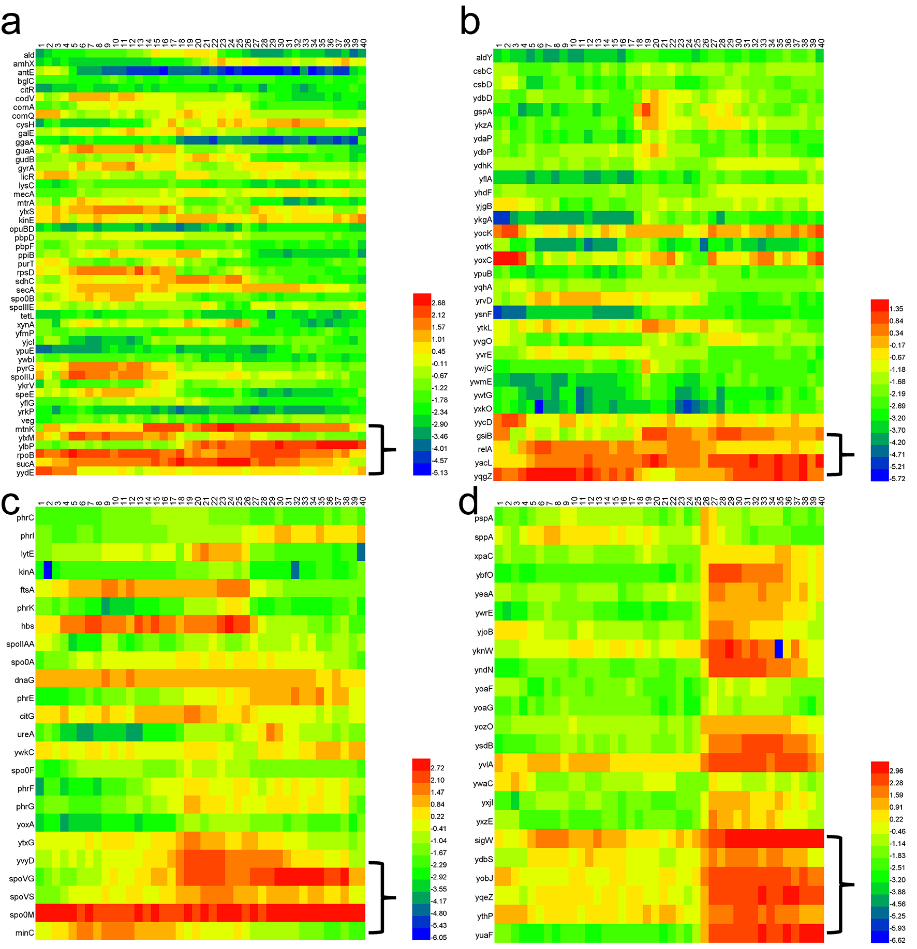


Figure S1 Transcriptional level analysis of sigma factor dependent promoters. Transcriptional levels of endogenous genes driven by σ^A^ (a), σ^B^ (b), σ^H^ (c) and σ^W^ (d) dependent constitutive promoters from *B.subtilis* 168 were analyzed and showed as heatmap. These data were obtained from Gene Expression Omnibus (GEO) of National Center for Biotechnology Information Search database (NCBI) (GEO accession: GSE19831)^1^. Names of genes were labeled on the left of figures, and the serial numbers above means various growth time of samples. The candidate promoters for further verification were marked by black brace.


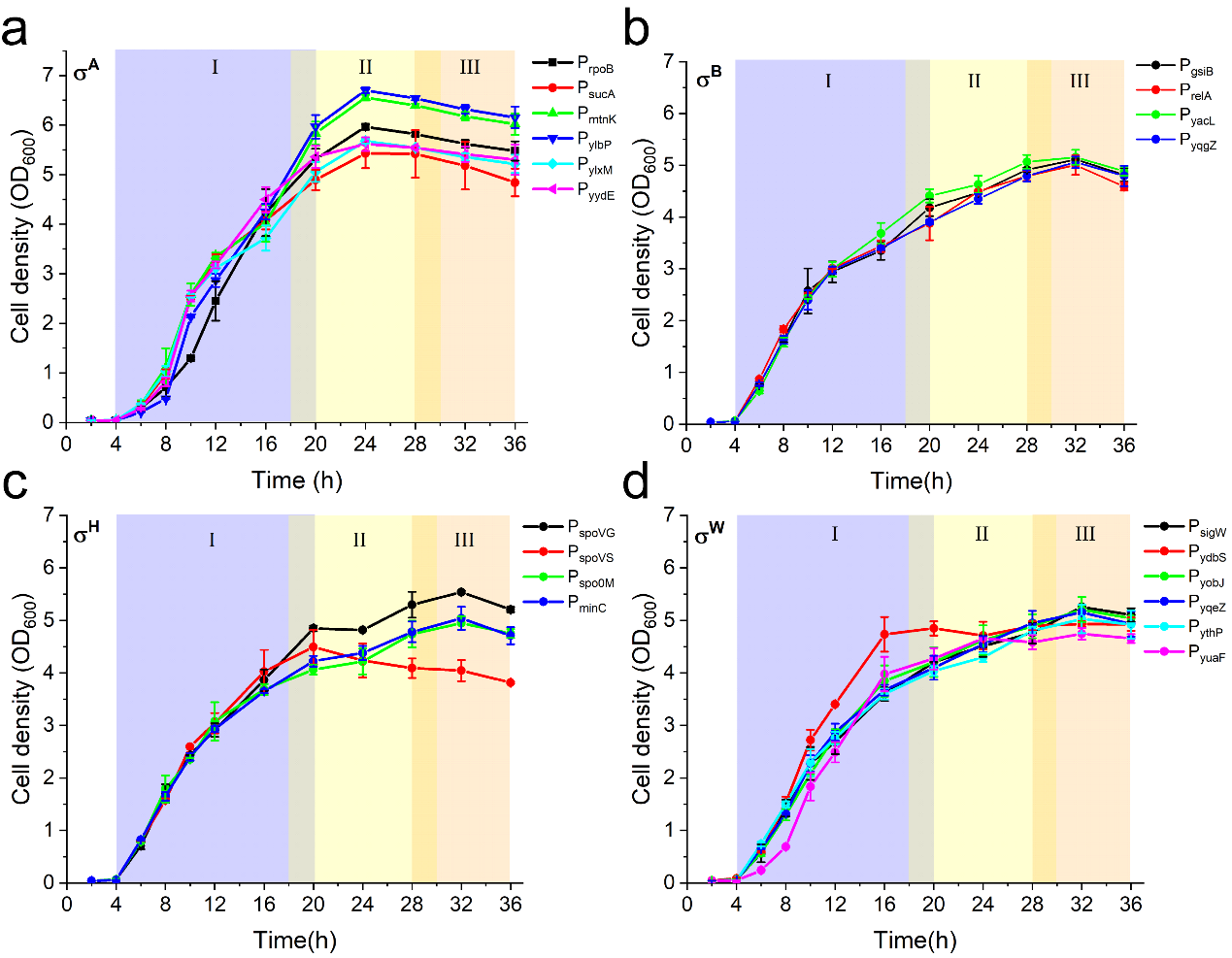


Figure S2 Growth curve of strains harboring single promoters screening plasmids. Candidate promoters recognized by σ^A^ (a), σ^B^ (b), σ^H^ (c) and σ^W^ (d) were grouped into four graphs. I: exponential growth phase (blue), II: early stationary phase (yellow), III: late stationary phase (orange). Error bar means the standard deviation of independent repeats in triplicate.





Figure S3 Determination of the specific recognition of single promoters by sigma factors. Plasmids harboring promoters recognized by σ^B^, σ^H^ and σ^W^ were transformed into wild type host *B.subtilis* 168 and corresponding sigma factors deficient host, then the GFP expression was determined to reflect promoters’ activity. Error bars are the standard deviation of three independent experiments.


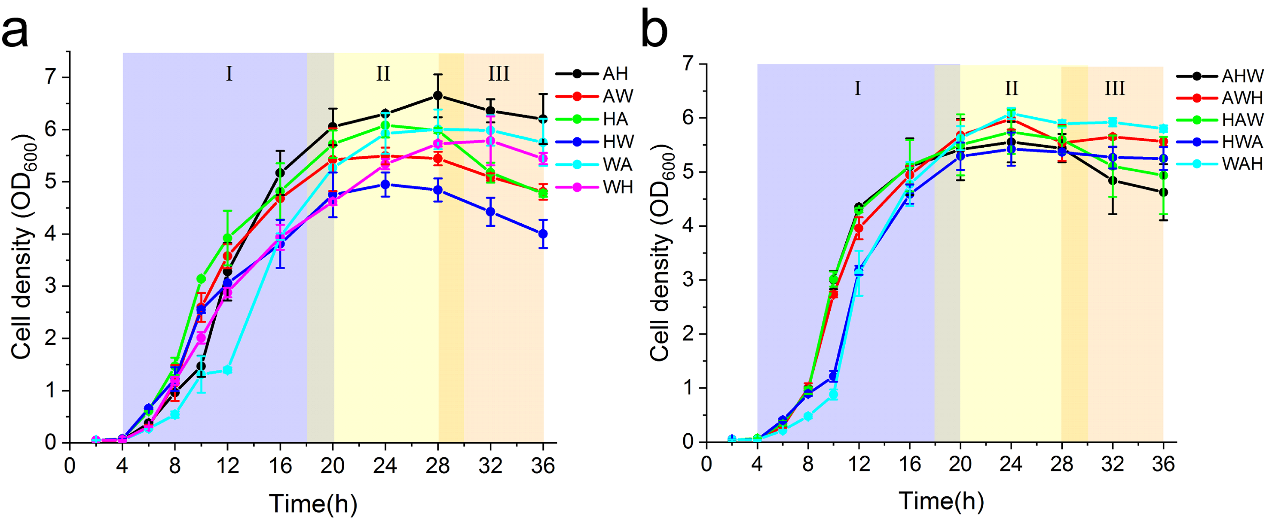


Figure S4 Growth curve of strains harboring GFP expression plasmids of dual promoters (a) and triple promoters (b). I: exponential growth phase (blue), II: early stationary phase (yellow), III: late stationary phase (orange). Error bar means the standard deviation of independent repeats in triplicate.


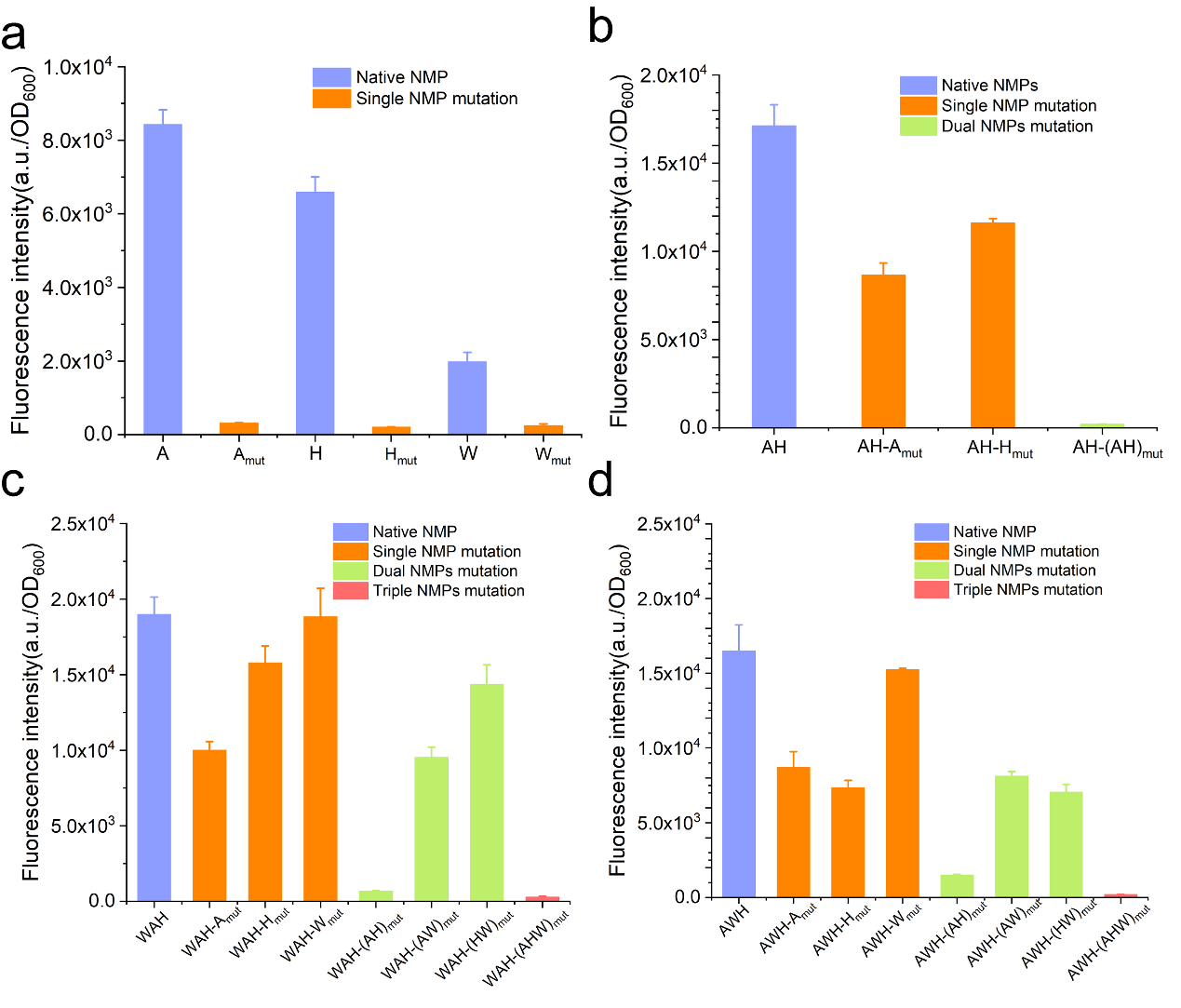


Figure S5 Determination of activity of AHPs and inactive AHPs. The Inactivating mutation of various NMP combinations was carried out to evaluate the contribution of each NMP to the activity of NMPs (a) and AH (b), WAH (c) and AWH (d). Error bars are the standard deviation of three independent experiments.


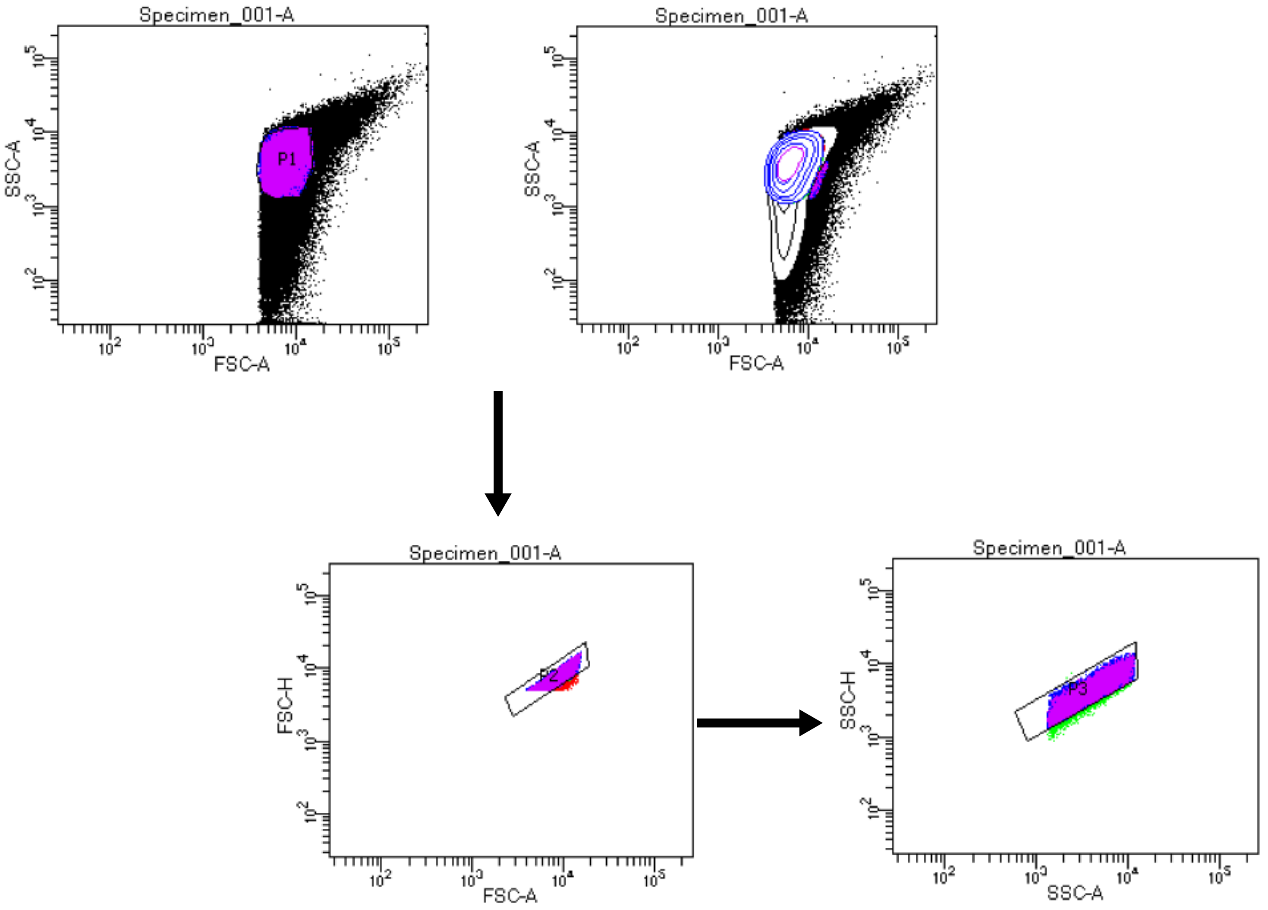


Figure S6 Gating strategy for flow cytometry analysis. Cell populations were gated according to their forward scatter (FSC) and side scatter (SSC) distributions to analyze the positive single cells.

Table S1 Plasmids and strains

| Plasmids and strains | Relevant characteristics | References or source |
| --- | --- | --- |
| Plasmids |  |  |
| pBSG03 | *E.coli*-*B.subtilis* shuttle vector, P*_srfA_*, gfp, Amp^R^ in *Escherichia coli*, Kan^R^ in *Bacillus subtilis* | Lab Stock^2^ |
| pHT01 | *E.coli*-*B.subtilis* shuttle vector, IPTG induced P*_grac_*, Amp^R^ in *Escherichia coli*, Cm^R^ in *Bacillus subtilis* | Lab Stock^3^ |
| pAX01 | *B.subtilis* integration vector, *xyl*^R^-P*_xyl_* cassette, Amp^R^ , Erm^R^ | Lab Stock |
| pT7-agrBD-I | Amp^R^, P_T7-lac_-*agr*B-*agr*D cassette | Addgene^4^ |
| pXylA-agrCA-I | *E.coli*-*B.subtilis* shuttle vector, *xyl*^R^-P*_xyl_* cassette, *agr*C, *agr*A, Amp^R^ in *Escherichia coli*, Cm^R^ in *Bacillus subtilis* | Addgene^4^ |
| pP3-GFP | *E.coli*-*B.subtilis* shuttle vector, P3-cycle3 GFP cassette, Amp^R^ in *Escherichia coli*, Tet^R^ in *Bacillus subtilis* | Addgene^4^ |
| pBRBS504-sfGFP | Derived from pBSG03, P*_srfA_* was deleted, gfp was substituted for RBS504-sfGFP fragment | This study |
| pBPrpoB-sfGFP | Derived from pBRBS504-sfGFP, sfGFP expressed by P*_rpoB_* | This study |
| pBPylbP-sfGFP | Derived from pBRBS504-sfGFP, sfGFP expressed by P*_ylbP_* | This study |
| pBPsucA-sfGFP | Derived from pBRBS504-sfGFP, sfGFP expressed by P*_sucA_* | This study |
| pBPylxM-sfGFP | Derived from pBRBS504-sfGFP, sfGFP expressed by P*_ylxM_* | This study |
| pBPyydE-sfGFP | Derived from pBRBS504-sfGFP, sfGFP expressed by P*_yydE_* | This study |
| pBPmtnK-sfGFP | Derived from pBRBS504-sfGFP, sfGFP expressed by P*_mtnK_* | This study |
| pBPgsiB-sfGFP | Derived from pBRBS504-sfGFP, sfGFP expressed by P*_gsiB_* | This study |
| pBPrelA-sfGFP | Derived from pBRBS504-sfGFP, sfGFP expressed by P*_relA_* | This study |
| pBPyacL-sfGFP | Derived from pBRBS504-sfGFP, sfGFP expressed by P*_yacL_* | This study |
| pBPyqgZ-sfGFP | Derived from pBRBS504-sfGFP, sfGFP expressed by P*_yqgZ_* | This study |
| pBPminC-sfGFP | Derived from pBRBS504-sfGFP, sfGFP expressed by P*_minC_* | This study |
| pBPspoVG-sfGFP | Derived from pBRBS504-sfGFP, sfGFP expressed by P*_spoVG_* | This study |
| pBPspoVS-sfGFP | Derived from pBRBS504-sfGFP, sfGFP expressed by P*_spoVS_* | This study |
| pBPspo0M-sfGFP | Derived from pBRBS504-sfGFP, sfGFP expressed by P*_spo0M_* | This study |
| pBPsigW-sfGFP | Derived from pBRBS504-sfGFP, sfGFP expressed by P*_sigW_* | This study |
| pBPydbS-sfGFP | Derived from pBRBS504-sfGFP, sfGFP expressed by P*_ydbS_* | This study |
| pBPyobJ-sfGFP | Derived from pBRBS504-sfGFP, sfGFP expressed by P*_yobJ_* | This study |
| pBPyqeZ-sfGFP | Derived from pBRBS504-sfGFP, sfGFP expressed by P*_yqeZ_* | This study |
| pBPythP-sfGFP | Derived from pBRBS504-sfGFP, sfGFP expressed by P*_ythP_* | This study |
| pBPyuaF-sfGFP | Derived from pBRBS504-sfGFP, sfGFP expressed by P*_yuaF_* | This study |
| pBPAH-sfGFP | Derived from pBPspoVG-sfGFP, P_rpoB_ was inserted into the upstream of P_spoVG_ | This study |
| pBPAW-sfGFP | Derived from pBPsigW-sfGFP, P_rpoB_ was inserted into the upstream of P_sigW_ | This study |
| pBPHA-sfGFP | Derived from pBPrpoB-sfGFP, P_spoVG_ was inserted into the upstream of P_rpoB_ | This study |
| pBPHW-sfGFP | Derived from pBPsigW-sfGFP, P_spoVG_ was inserted into the upstream of P_sigW_ | This study |
| pBPWA-sfGFP | Derived from pBPrpoB-sfGFP, P_rpoB_ was inserted into the upstream of P_spoVG_ | This study |
| pBPWH-sfGFP | Derived from pBPspoVG-sfGFP, P_sigW_ was inserted into the upstream of P_spoVG_ | This study |
| pBPAHW-sfGFP | Derived from pBPHW-sfGFP, P_rpoB_ was inserted into the upstream of HW | This study |
| pBPAWH-sfGFP | Derived from pBPWH-sfGFP, P_rpoB_ was inserted into the upstream of WH | This study |
| pBPHAW-sfGFP | Derived from pBPAW-sfGFP, P_spoVG_ was inserted into the upstream of AW | This study |
| pBPHWA-sfGFP | Derived from pBPWA-sfGFP, P_spoVG_ was inserted into the upstream of WA | This study |
| pBPWAH-sfGFP | Derived from pBPAH-sfGFP, P_sigW_ was inserted into the upstream of AH | This study |
| pBPWHA-sfGFP | Derived from pBPHA-sfGFP, P_sigW_ was inserted into the upstream of HA | This study |
| pBPrpoB_mut_-sfGFP | Derived from pBPrpoB-sfGFP, -10 box of P*_rpoB_* was mutated | This study |
| pBPspoVG_mut_-sfGFP | Derived from pBPspoVG-sfGFP, -10 box of P*_spoVG_* was mutated | This study |
| pBPsigW_mut_-sfGFP | Derived from pBPsigW-sfGFP, -10 box of P*_sigW_* was mutated | This study |
| pBPAH-A_mut_-sfGFP | Derived from pBPAH-sfGFP, -10 box of P*_rpoB_* was mutated | This study |
| pBPAH-H_mut_-sfGFP | Derived from pBPAH-sfGFP, -10 box of P*_spoVG_* was mutated | This study |
| pBPAH-AH_mut_-sfGFP | Derived from pBPAH-sfGFP, -10 boxes of P*_rpoB_* and P*_spoVG_* were mutated simultaneously | This study |
| pBPAWH-A_mut_-sfGFP | Derived from pBPAWH-sfGFP, -10 box of P*_rpoB_* was mutated | This study |
| pBPAWH-H_mut_-sfGFP | Derived from pBPAWH-sfGFP, -10 box of P*_spoVG_* was mutated | This study |
| pBPAWH-W_mut_-sfGFP | Derived from pBPAWH-sfGFP, -10 box of P*_sigW_* was mutated | This study |
| pBPAWH-AH_mut_-sfGFP | Derived from pBPAWH-sfGFP, -10 boxes of P*_rpoB_* and P*_spoVG_* were mutated simultaneously | This study |
| pBPAWH-AW_mut_-sfGFP | Derived from pBPAWH-sfGFP, -10 boxes of P*_rpoB_* and P*_sigW_* were mutated simultaneously | This study |
| pBPAWH-HW_mut_-sfGFP | Derived from pBPAWH-sfGFP, -10 boxes of P*_spoVG_* and P*_sigW_* were mutated simultaneously | This study |
| pBPAWH-AHW_mut_-sfGFP | Derived from pBPAWH-sfGFP, -10 boxes of P*_rpoB_*,P*_spoVG_* and P*_sigW_* were mutated simultaneously | This study |
| pBPWAH-A_mut_-sfGFP | Derived from pBPWAH-sfGFP, -10 box of P*_rpoB_* was mutated | This study |
| pBPWAH-H_mut_-sfGFP | Derived from pBPWAH-sfGFP, -10 box of P*_spoVG_* was mutated | This study |
| pBPWAH-W_mut_-sfGFP | Derived from pBPWAH-sfGFP, -10 box of P*_sigW_* was mutated | This study |
| pBPWAH-AH_mut_-sfGFP | Derived from pBPWAH-sfGFP, -10 boxes of P*_rpoB_* and P*_spoVG_* were mutated simultaneously | This study |
| pBPWAH-AW_mut_-sfGFP | Derived from pBPWAH-sfGFP, -10 boxes of P*_rpoB_* and P*_sigW_* were mutated simultaneously | This study |
| pBPWAH-HW_mut_-sfGFP | Derived from pBPWAH-sfGFP, -10 boxes of P*_spoVG_* and P*_sigW_* were mutated simultaneously | This study |
| pBPWAH-AHW_mut_-sfGFP | Derived from pBPWAH-sfGFP, -10 boxes of P*_rpoB_*, P*_spoVG_* and P*_sigW_* were mutated simultaneously | This study |
| pBPAH-Dn-sfGFP | Spacer sequences of various length were inserted between core regions of dual promoter P_AH_ | This study |
| pBPAWH-Dn-sfGFP | Spacer sequences of various length were inserted between core regions of P*_sigW_* and P*_spoVG_* in P_AWH_ | This study |
| pBPAWH-Un-sfGFP | Spacer sequences of various length were inserted between core regions of P*_rpoB_* and P*_sigW_* in P_AWH_ | This study |
| pBPWAH-Dn-sfGFP | Spacer sequences of various length were inserted between core regions of P*_spoB_* and P*_spoVG_* in P_WAH_ | This study |
| pBPWAH-Un-sfGFP | Spacer sequences of various length were inserted between core regions of P*_sigW_* and P*_rpoB_* in P_WAH_ | This study |
| pBP_x_-RBS_n_-sfGFP | gfp expressed by various combinations of promoters and RBSs | This study |
| pHT-PAWH-sfGFP | Derived from pHT01, Pgrac was substituted for P_AWHD30_-RBS106-sfGFP fragment | This study |
| pHT-PAWH-lac-sfGFP | Derived from pHT01, Pgrac was substituted for P_AWHD30_-lacO-RBS106- sfGFP fragment | This study |
| pHT-PAWH-xyl-sfGFP | Derived from pHT01, Pgrac was substituted for P_AWHD30_-xylO-RBS106-sfGFP fragment, lacI was substituted for xylR | This study |
| pHT-PAWH-lac-Pul | Derived from pHT-PAWH-lac-sfGFP, sfGFP was substituted for pulWB42 gene | This study |
| pHT-PAWH-xyl-agr-106 | Derived from pHT-PAWH-xyl-sfGFP, agrD expression was controlled by Promoter P_AWH-D30_ and RBS106, sfGFP expression was driven by P3 | This study |
| pHT-PAWH-xyl-agr-503 | Derived from pHT-PAWH-xyl-sfGFP, agrD expression was controlled by Promoter P_AWH-D30_ and RBS503, sfGFP expression was driven by P3 | This study |
| pAX-P43-agrBCA | Derived from pAX01, P43-agrB-agrC-agrA expression cassette was cloned into pAX01 | This study |
| p7Z6 | pMD18-T containing lox71-zeo-lox66 cassette | Lab Stock^5^ |
| psigB-del | Derived from p7z6, containing homologous arm for sigB knock out | This study |
| psigH-del | Derived from p7z6, containing homologous arm for sigH knock out | This study |
| psigW-del | Derived from p7z6, containing homologous arm for sigW knock out | This study |
| Strains |  |  |
| *E.coli* JM109 | *recA*1, *supE*44 *endA*1 *hsdR*17 ( ^r-^k,m^+^ k) *gyrA*96 *relA*1 thi (*lac-proAB*) F’[*traD*36 *proAB*^+^ *lacI*^q^ *lacZ* ΔM15] | Lab Stock |
| *B.subtilis* 168 | *trpC*2 | Lab Stock |
| *B.subtilis* 168 (△sigB) | Derived from *B.subtilis* 168, sigB was knocked out | This study |
| *B.subtilis* 168 (△sigH) | Derived from *B.subtilis* 168, sigH was knocked out | This study |
| *B.subtilis* 168 (△sigW) | Derived from *B.subtilis* 168, sigW was knocked out | This study |
| BsBCA | Derived from *B.subtilis* 168, erm-P43-agrB-agrC-agrA fragment was integrated into the lacA site by homologous recombination | This study |

Table S2 Primers

| Primers | Sequences (5’-3’) |
| --- | --- |
| PrpoB-1 | CGGTATTTTAACTATGTTAATATTGTAAAATGCCAATGTATTCGAACATCATATTTAAAGTACGAGGAG |
| PrpoB-2 | ACAATATTAACATAGTTAAAATACCGAGTCAAACTTTTTTTGCTTACCTGCCCTCTGCCACC |
| PsfGFP-i1 | CATCATATTTAAAGTACGAGGAGTCCACATatgagcaaaggagaagaacttttcac |
| PsfGFP-i2 | AGGCGAATTCTTAtttgtagagctcatccatgcc |
| PsfGFP-v1 | gagctctacaaaTAAGAATTCGCCTGATGCG |
| PsfGFP-v2 | GACTCCTCGTACTTTAAATATGATGTTCGAACCTGCCCTCTGCCACC |
| P_TH_-1 | CTCCTGTACTAGGAGGGTATTTTTTTTTTCTCCTTACGCATCTGTGC |
| P_TH_-2 | AAAAAATACCCTCCTAGTACAGGAGGGATACCGCATCAGGCGAATTC |
| PsucA-1 | ACAATCAAGGTAGAATCAAATTGCAAACAGTGGTAAAATATTCGAACATCATATTTAAAGTACGAGGAG |
| PsucA-2 | TTGCAATTTGATTCTACCTTGATTGTTCACAAAATAGTAAAAAACACCTGCCCTCTGCCACC |
| PylbP-1 | TTTTTTAAATAAAGCGTTTACAATATATGTAGAAACAACAATCGAACATCATATTTAAAGTACGAGGAG |
| PylbP-2 | ATATTGTAAACGCTTTATTTAAAAAATCCAAATATTTAAACTTTAACCTGCCCTCTGCCACC |
| PylxM-1 | GTGTCATTAAAACCGTGTAAACTAAGTTATCGTAAAGGGATTCGAACATCATATTTAAAGTACGAGGAG |
| PylxM-2 | CTTAGTTTACACGGTTTTAATGACACTGTCAAGTTTTTATCTTGTACCTGCCCTCTGCCACC |
| PyydE-1 | AAAGCAGTTATGCGGTACTATCATATAAAGGTCCAATGTTTTCGAACATCATATTTAAAGTACGAGGAG |
| PyydE-2 | ATATGATAGTACCGCATAACTGCTTTTAGAGACAATTAAAACGAGACCTGCCCTCTGCCACC |
| PmtnK-1 | CTAACTAAATTACCTGTTACCATGTTCATCAACTGATAAATTCGAACATCATATTTAAAGTACGAGGAG |
| PmtnK-2 | AACATGGTAACAGGTAATTTAGTTAGTTGTCAATATATTTTTTAAACCTGCCCTCTGCCACC |
| PgsiB-1 | TTTAAAAGAATTGTGAGCGGGAATACAACAACCAACACCAATCGAACATCATATTTAAAGTACGAGGAG |
| PgsiB-2 | GTATTCCCGCTCACAATTCTTTTAAACAAACAAAACACATTGGGTACCTGCCCTCTGCCACC |
| PrelA-1 | TTTTATATAATATTTGGCTATTTGAACTTCTGCTCTTTACATCGAACATCATATTTAAAGTACGAGGAG |
| PrelA-2 | TTCAAATAGCCAAATATTATATAAAATAAATGCAAAACAATAGGAACCTGCCCTCTGCCACC |
| PyacL-1 | AAACCTTATGAATACGGGTATATTAATGTTGGTTTTTGTTTTCGAACATCATATTTAAAGTACGAGGAG |
| PyacL-2 | TTAATATACCCGTATTCATAAGGTTTTAACCGAAATCACAGTTGTACCTGCCCTCTGCCACC |
| PyqgZ-1 | GAAAAATGATCCGGGTAGTTATTCTACAAAGAGATGGTTAATCGAACATCATATTTAAAGTACGAGGAG |
| PyqgZ-2 | TAGAATAACTACCCGGATCATTTTTCATTTAAACCATTTACAGAAACCTGCCCTCTGCCACC |
| PminC-1 | GATTTTATCTTTTTTTGACGAAATGAGTATGTTGTTGAGGTTCGAACATCATATTTAAAGTACGAGGAG |
| PminC-2 | TCATTTCGTCAAAAAAAGATAAAATCCTTTTTACTCATCTCTCAAACCTGCCCTCTGCCACC |
| PspoVG-1 | TTTCAGAAAAAATCGTGGAATTGATACACTAATGCTTTTATTCGAACATCATATTTAAAGTACGAGGAG |
| PspoVG-2 | TATCAATTCCACGATTTTTTCTGAAATCCTGCTCGTTTTTAAAATACCTGCCCTCTGCCACC |
| PspoVS-1 | GAATATAGCAACTCCTTAGTGAATATAGTAAAAATGGAAGGTCGAACATCATATTTAAAGTACGAGGAG |
| PspoVS-2 | ATATTCACTAAGGAGTTGCTATATTCCTGCTTTTCTTTTTAATATACCTGCCCTCTGCCACC |
| Pspo0M-1 | GAAAAAAGTATGAATCAAACGAATCTTTTTTCCTCCTTCTTTCGAACATCATATTTAAAGTACGAGGAG |
| Pspo0M-2 | AGATTCGTTTGATTCATACTTTTTTCCTATTATTCGTCTCGGCCTACCTGCCCTCTGCCACC |
| PsigW-1 | ACCTTTTGAAACGAAGCTCGTATACATACAGACCGGTGAAGTCGAACATCATATTTAAAGTACGAGGAG |
| PsigW-2 | TGTATACGAGCTTCGTTTCAAAAGGTTTCAATTTTTTTATAAAATACCTGCCCTCTGCCACC |
| PydbS-1 | ACCTTTCTGTAAAAGAGACGTATAAATAACGACGAAAAAAATCGAACATCATATTTAAAGTACGAGGAG |
| PydbS-2 | TTTATACGTCTCTTTTACAGAAAGGTTTCATTCTTAAGCATACAGACCTGCCCTCTGCCACC |
| PyobJ-1 | ACCTTTTTTATTTTAGCCCGTATTAAAAGTAAATTCAGAGATCGAACATCATATTTAAAGTACGAGGAG |
| PyobJ-2 | TTAATACGGGCTAAAATAAAAAAGGTTTCATATAAAACGGGACTAACCTGCCCTCTGCCACC |
| PyqeZ-1 | AACCTTTGATACATTTGTTACGTATGAAGAGAAGGCACTTATCGAACATCATATTTAAAGTACGAGGAG |
| PyqeZ-2 | CATACGTAACAAATGTATCAAAGGTTTCATTTTTTTATGTATAAAACCTGCCCTCTGCCACC |
| PythP-1 | AAACTTTTTTTATTCTATTTCGTAGTAAATTTTGGAGGTGATCGAACATCATATTTAAAGTACGAGGAG |
| PythP-2 | ACTACGAAATAGAATAAAAAAAGTTTCTTTAACCATAATAATATTACCTGCCCTCTGCCACC |
| PyuaF-1 | ACTTTTCCCGAGGTGTCTCGTATAAATGGTAACGGCAGCCGTCGAACATCATATTTAAAGTACGAGGAG |
| PyuaF-2 | TTTATACGAGACACCTCGGGAAAAGTTTCAAAATTTTAAGACAAAACCTGCCCTCTGCCACC |
| PrpoB-spoVG-1 | CGGTATTTTAACTATGTTAATATTGTAAAATGCCAATGTATATTTTAAAAACGAGCAGGATTTCAG |
| PrpoB-sigW-1 | CGGTATTTTAACTATGTTAATATTGTAAAATGCCAATGTATATTTTATAAAAAAATTGAAACCTTTTGAAAC |
| PspoVG-rpoB-1 | TTTCAGAAAAAATCGTGGAATTGATACACTAATGCTTTTATAAGCAAAAAAAGTTTGACTCG |
| PspoVG-sigW-1 | TTTCAGAAAAAATCGTGGAATTGATACACTAATGCTTTTATATTTTATAAAAAAATTGAAACCTTTTGAAACG |
| PsigW-rpoB-1 | ACCTTTTGAAACGAAGCTCGTATACATACAGACCGGTGAAGAAGCAAAAAAAGTTTGACTCG |
| PsigW-spoVG-1 | ACCTTTTGAAACGAAGCTCGTATACATACAGACCGGTGAAGATTTTAAAAACGAGCAGGATTTCAG |
| PrpoB-mut-1 | TTAACTATGTGCCGCGTGTAAAATGCCAATGTAT |
| PrpoB-mut-2 | CATTTTACACGCGGCACATAGTTAAAATACCGAGTC |
| PspoVG-mut-1 | AAAAATCGTGTCCGTGATACACTAATGCTTTTAT |
| PspoVG-mut-2 | TTAGTGTATCACGGACACGATTTTTTCTGAAATCC |
| PsigW-mut-1 | AACGAAGCTATGCGCCATACAGACCGGTGAAG |
| PsigW-mut-2 | GGTCTGTATGGCGCATAGCTTCGTTTCAAAAGGTTTC |
| PWAH-D15-1 | AAAATGCCAATGTATATTTTTTCAAAAAATATTTTAAAAACGAGC |
| PWAH-D15-2 | ATTTTTTGAAAAAATATACATTGGCATTTTACAATATTAAC |
| PWAH-D30-1 | GTGTAACTATATCCTATTTTTTCAAAAAATATTTTAAAAACGAGCAGGATTTC |
| PWAH-D30-2 | ATTTTTTGAAAAAATAGGATATAGTTACACATACATTGGCATTTTACAATATTAAC |
| PWAH-D45-1 | AAAATGCCAATGTATCTTTATGACCTAATTGTGTAACTATATCC |
| PWAH-D45-2 | AATTAGGTCATAAAGATACATTGGCATTTTACAATATTAAC |
| PWAH-D60-1 | TGCCCGAAATGAAAGCTTTATGACCTAATTGTGTAACTATATCCTATTTTTTC |
| PWAH-D60-2 | AATTAGGTCATAAAGCTTTCATTTCGGGCAATACATTGGCATTTTACAATATTAAC |
| PWAH-D75-1 | TGTATTTTTTATTTACCTTATGCCCGAAATGAAAGCTTTATG |
| PWAH-D75-2 | ATTTCGGGCATAAGGTAAATAAAAAATACATTGGCATTTTACAATATTAAC |
| PWAH-D90-1 | AGAGCCGGGATCACTTTTTTATTTACCTTATGCCCGAAATGAAAGCTTTATG |
| PWAH-D90-2 | TAAGGTAAATAAAAAAGTGATCCCGGCTCTATACATTGGCATTTTACAATATTAAC |
| PWAH-U15-1 | CGGTGAAGGCTATTATATCATAAAAGCAAAAAAAGTTTGACTCG |
| PWAH-U15-2 | TTTGCTTTTATGATATAATAGCCTTCACCGGTCTGTATGTATAC |
| PWAH-U30-1 | TTCAAAAAAAGAAAGGCTATTATATCATAAAAGCAAAAAAAGTTTGACTCG |
| PWAH-U30-2 | TTATGATATAATAGCCTTTCTTTTTTTGAACTTCACCGGTCTGTATGTATAC |
| PWAH-U45-1 | CGGTGAAGATGAAGTTTCCGTCGTTCAAAAAAAGAAAGGCTATTATATC |
| PWAH-U45-2 | TTTTGAACGACGGAAACTTCATCTTCACCGGTCTGTATGTATAC |
| PWAH-U60-1 | TCGAGGAACTGTTCGATGAAGTTTCCGTCGTTCAAAAAAAGAAAGGCTATTATATC |
| PWAH-U60-2 | CGACGGAAACTTCATCGAACAGTTCCTCGACTTCACCGGTCTGTATGTATAC |
| PWAH-U75-1 | CGGTGAAGCTGCCATTGAAAAGCTCGAGGAACTGTTCGATG |
| PWAH-U75-2 | TCCTCGAGCTTTTCAATGGCAGCTTCACCGGTCTGTATGTATAC |
| PWAH-U90-1 | AGCAAGGCGCGCCTTCTGCCATTGAAAAGCTCGAGGAACTGTTCGATG |
| PWAH-U90-2 | GCTTTTCAATGGCAGAAGGCGCGCCTTGCTCTTCACCGGTCTGTATGTATAC |
| PAWH-D15-1 | TACAGACCGGTGAAGATTTTTTCAAAAAATATTTTAAAAACGAGC |
| PAWH-D15-2 | ATTTTTTGAAAAAATCTTCACCGGTCTGTATGTATAC |
| PAWH-D30-2 | ATTTTTTGAAAAAATAGGATATAGTTACACCTTCACCGGTCTGTATGTATAC |
| PAWH-D45-1 | TACAGACCGGTGAAGCTTTATGACCTAATTGTGTAACTATATCC |
| PAWH-D45-2 | AATTAGGTCATAAAGCTTCACCGGTCTGTATGTATAC |
| PAWH-D60-2 | AATTAGGTCATAAAGCTTTCATTTCGGGCACTTCACCGGTCTGTATGTATAC |
| PAWH-D75-1 | TGAAGTTTTTATTTACCTTATGCCCGAAATGAAAGCTTTATG |
| PAWH-D75-2 | ATTTCGGGCATAAGGTAAATAAAAACTTCACCGGTCTGTATGTATAC |
| PAWH-90-2 | TAAGGTAAATAAAAAAGTGATCCCGGCTCTCTTCACCGGTCTGTATGTATAC |
| PAWH-U15-1 | AAAATGCCAATGTATAAATTTGATAAACTTATTTTATAAAAAAATTGAAACC |
| PAWH-U15-2 | AAGTTTATCAAATTTATACATTGGCATTTTACAATATTAAC |
| PAWH-U30-1 | TCTACACCCTGCCAAAAATTTGATAAACTTATTTTATAAAAAAATTGAAACCTTTTG |
| PAWH-U30-2 | AAGTTTATCAAATTTTTGGCAGGGTGTAGAATACATTGGCATTTTACAATATTAAC |
| PAWH-U45-1 | AAAATGCCAATGTATATTCACGAATTACCATCTACAC |
| PAWH-U45-2 | TGGTAATTCGTGAATATACATTGGCATTTTACAATATTAAC |
| PAWH-U60-1 | ATGTTAAGGTAGTTTATTCACGAATTACCATCTACACCCTGCCAAAAATTTG |
| PAWH-U60-2 | TGGTAATTCGTGAATAAACTACCTTAACATATACATTGGCATTTTACAATATTAAC |
| PAWH-U75-1 | TGTATAATAATTTTAAAAATATGTTAAGGTAGTTTATTCACGAATTACC |
| PAWH-U75-2 | ACCTTAACATATTTTTAAAATTATTATACATTGGCATTTTACAATATTAAC |
| PAWH-U90-1 | CTGTTCAGTCTGTATAATAATTTTAAAAATATGTTAAGGTAGTTTATTCACGAATTACC |
| PAWH-U90-2 | ATTTTTAAAATTATTATACAGACTGAACAGATACATTGGCATTTTACAATATTAAC |
| PAWH-DU30-1 | TCTACACCCTGCCAAAAATTTGATAAACTTATTTTAAAAACGAGCAGGATTTC |
| PAWH-DU30-2 | AAGTTTATCAAATTTTTGGCAGGGTGTAGACTTCACCGGTCTGTATGTATAC |
| Prbs103-1 | TATTTAAAGTGCCGCTACACCACATATGAGCAAAGGAGAAG |
| Prbs103-2 | ATGTGGTGTAGCGGCACTTTAAATATGATGTTCGA |
| Prbs503-1 | TATTTAAAGTCGGAGTACTCCACATATGAGCAAAGGAGAAG |
| Prbs503-2 | ATGTGGAGTACTCCGACTTTAAATATGATGTTCGA |
| Prbs104-1 | TATTTAAAGTAGGTTCGTTCCACATATGAGCAAAGGAGAAG |
| Prbs104-2 | ATGTGGAACGAACCTACTTTAAATATGATGTTCGA |
| Prbs304-1 | TATTTAAAGTAAAGGTTACCCACATATGAGCAAAGGAGAAG |
| Prbs304-2 | ATGTGGGTAACCTTTACTTTAAATATGATGTTCGA |
| Prbs804-1 | TATTTAAAGTAATAGGAGTCCACATATGAGCAAAGGAGAAG |
| Prbs804-2 | ATGTGGACTCCTATTACTTTAAATATGATGTTCGA |
| Prbs105-1 | TATTTAAAGTCGAGGTAAACCACATATGAGCAAAGGAGAAG |
| Prbs105-2 | ATGTGGTTTACCTCGACTTTAAATATGATGTTCGA |
| Prbs305-1 | TATTTAAAGTAAAGGAGTCCCACATATGAGCAAAGGAGAAG |
| Prbs305-2 | ATGTGGGACTCCTTTACTTTAAATATGATGTTCGA |
| Prbs505-1 | TATTTAAAGTAGAGGAGGGCCACATATGAGCAAAGGAGAAG |
| Prbs505-2 | ATGTGGCCCTCCTCTACTTTAAATATGATGTTCGA |
| Prbs805-1 | TATTTAAAGTAAGGAGGTTCCACATATGAGCAAAGGAGAAG |
| Prbs805-2 | ATGTGGAACCTCCTTACTTTAAATATGATGTTCGA |
| Prbs106-1 | TATTTAAAGTACAGGAGGTCCACATATGAGCAAAGGAGAAG |
| Prbs106-2 | ATGTGGACCTCCTGTACTTTAAATATGATGTTCGA |
| Prbs156-1 | TATTTAAAGTCAAGGAGGTCCACATATGAGCAAAGGAGAAG |
| Prbs156-2 | ATGTGGACCTCCTTGACTTTAAATATGATGTTCGA |
| Prbs206-1 | TATTTAAAGTAAAGGAGGTCCACATATGAGCAAAGGAG |
| Prbs206-2 | ATGTGGACCTCCTTTACTTTAAATATGATGTTCGA |
| PpHT-AWH-i1 | ctaacggaaaagggaTTTTTGAGTGATCTTCTCAAAAAATAC |
| PpHT-AWH-i2 | cctcgtatgtttcaaAGAGTGCACCATATGCGG |
| PpHT-AWH-v1 | CATATGGTGCACTCTttgaaacatacgaggctaatatcgg |
| PpHT-AWH-v2 | AAGATCACTCAAAAAtcccttttccgttagctttttc |
| PAWH-lacO-1 | ggaattgtgagcggataacaattccATGCTTTTATTCGAACATCATATTTAAAG |
| PAWH-lacO-2 | ggaattgttatccgctcacaattccTAGTGTATCAATTCCACGATTTTTTC |
| PAWH-xylO-1 | AGTTAGTTTATTGGATAAACAAACTAACTATGCTTTTATTCGAACATCATATTTAAAG |
| PAWH-xylO-2 | AGTTAGTTTGTTTATCCAATAAACTAACTTAGTGTATCAATTCCACGATTTTTTC |
| PAWH-xylR-i1 | ttaattgcgttgcgcCTAACTTATAGGGGTAACACTTAAAAAAG |
| PAWH-xylR-i2 | agggagacgattttgATGGTTATTATTCAAATTGCAGATC |
| PAWH-xylR-v1 | TTGAATAATAACCATcaaaatcgtctccctcc |
| PAWH-xylR-v2 | ACCCCTATAAGTTAGgcgcaacgcaattaatg |
| PAWH-agrD-i1 | CACATATGAATACATTATTTAACTTATTTTTTGATTTTATTACTG |
| PAWH-agrD-i2 | TAAGGAGAAATTAATATTTTAACATAAAAAAATTTACAGTTAAG |
| PAWH-agrD-v1 | ATGTTAAAATATTAATTTCTCCTTACGCATCTGTGC |
| PAWH-agrD-v2 | AAGTTAAATAATGTATTCATATGTGGACCTCCTGTACTTTAAATATG |
| PAWH-agrD-503-v2 | AAGTTAAATAATGTATTCATATGTGGAGTACTCCGACTTTAAATATG |
| PAWH-Plu-i1 | ACAGGAGGTCCACATATGCTAACGGTTCATCGGACG |
| PAWH-Plu-i2 | AGGCGAATTCTTAAGTCAGTCCTTTCACAAGC |
| PAWH-Plu-v1 | AAAGGACTGACTTAAGAATTCGCCTGATGCGGTATC |
| PAWH-Plu-v2 | CCGTTAGCATATGTGGACCTCCTGTACTTTAAATATGATG |
| PlacA -1 | CTAATGTGTGTTTACGACAATTCTCACTTC |
| PlacA -2 | GTGATGTCAAAGCTTGAAAAAACGC |
| PsigB-del-F1 | ggaaagcgggcagtgTTGGGCGTTTTTGTAGGG |
| PsigB-del-F2 | ggtcatagctgtttcctgGATCAACTCGCTCCCC |
| PsigB-del-V1 | CTCGGACAGCTGTCCTAAActggcgtaatagcgaagagg |
| PsigB-del-V2 | CCCTACAAAAACGCCCAAcactgcccgctttcc |
| PsigB-del-B1 | GGGGAGCGAGTTGATCcaggaaacagctatgacc |
| PsigB-del-B2 | CGTTTTCTTCAACCTGGATCAgtaaaacgacggccagtg |
| PsigB-del-D1 | cactggccgtcgttttacTGATCCAGGTTGAAGAAAACG |
| PsigB-del-D2 | cctcttcgctattacgccagTTTAGGACAGCTGTCCGAG |
| PsigH-del-F1 | ggaaagcgggcagtgAAACCCTGTGGGAGAATAAAG |
| PsigH-del-F2 | ggtcatagctgtttcctgCGGCGCACGTAGATAG |
| PsigH-del-B1 | CTATCTACGTGCGCCGcaggaaacagctatgacc |
| PsigH-del-B2 | CCTATTACAAACTGATTTCGCGgtaaaacgacggccagtg |
| PsigH-del-D1 | cactggccgtcgttttacCGCGAAATCAGTTTGTAATAGG |
| PsigH-del-D2 | cctcttcgctattacgccagAGTGAATTACCATAACGCTTC |
| PsigH-del-V1 | GAAGCGTTATGGTAATTCACTctggcgtaatagcgaagagg |
| PsigW-del-F1 | cgcaaaccgcctctCACCATTTGGAGGATTAGC |
| PsigW-del-F2 | ccgagctcgaattcgtaATTTATCTAACCTCTGCCTTCAC |
| PsigW-del-D1 | gcaagcttggcactggGTGGGGTGATGAAATGAGC |
| PsigW-del-D2 | ggcgtatcacgaggcTTCCCCGCAGACTTCATC |
| PsigW-del-B1 | GTGAAGGCAGAGGTTAGATAAATtacgaattcgagctcgg |
| PsigW-del-B2 | GCTCATTTCATCACCCCACccagtgccaagcttgc |
| PsigW-del-v1 | GATGAAGTCTGCGGGGAAgcctcgtgatacgcc |
| PsigW-del-v2 | GCTAATCCTCCAAATGGTGagaggcggtttgcg |

Table S3 Sigma factors dependent natural minimal promoters selected for clone and characterization

| Promoter | Sequences (5’-3’) |
| --- | --- |
| P_rpoB_ | AAGCAAAAAAAGTTTGACTCGGTATTTTAACTATGTTAATATTGTAAAATGCCAATGTAT |
| P_sucA_ | GTTTTTTACTATTTTGTGAACAATCAAGGTAGAATCAAATTGCAAACAGTGGTAAAATAT |
| P_ylbP_ | TAAAGTTTAAATATTTGGATTTTTTAAATAAAGCGTTTACAATATATGTAGAAACAACAA |
| P_ylxM_ | ACAAGATAAAAACTTGACAGTGTCATTAAAACCGTGTAAACTAAGTTATCGTAAAGGGAT |
| P_yydE_ | CTCGTTTTAATTGTCTCTAAAAGCAGTTATGCGGTACTATCATATAAAGGTCCAATGTTT |
| P_mtnK_ | TTAAAAAATATATTGACAACTAACTAAATTACCTGTTACCATGTTCATCAACTGATAAAT |
| P_gsiB_ | ACCCAATGTGTTTTGTTTGTTTAAAAGAATTGTGAGCGGGAATACAACAACCAACACCAA |
| P_relA_ | TCCTATTGTTTTGCATTTATTTTATATAATATTTGGCTATTTGAACTTCTGCTCTTTACA |
| P_yacL_ | ACAACTGTGATTTCGGTTAAAACCTTATGAATACGGGTATATTAATGTTGGTTTTTGTTT |
| P_yqgZ_ | TTCTGTAAATGGTTTAAATGAAAAATGATCCGGGTAGTTATTCTACAAAGAGATGGTTAA |
| P_minC_ | TTGAGAGATGAGTAAAAAGGATTTTATCTTTTTTTGACGAAATGAGTATGTTGTTGAGGT |
| P_spoVG_ | ATTTTAAAAACGAGCAGGATTTCAGAAAAAATCGTGGAATTGATACACTAATGCTTTTAT |
| P_spoVS_ | ATATTAAAAAGAAAAGCAGGAATATAGCAACTCCTTAGTGAATATAGTAAAAATGGAAGG |
| P_spo0M_ | AGGCCGAGACGAATAATAGGAAAAAAGTATGAATCAAACGAATCTTTTTTCCTCCTTCTT |
| P_sigW_ | ATTTTATAAAAAAATTGAAACCTTTTGAAACGAAGCTCGTATACATACAGACCGGTGAAG |
| P_ydbS_ | CTGTATGCTTAAGAATGAAACCTTTCTGTAAAAGAGACGTATAAATAACGACGAAAAAAA |
| P_yobJ_ | TAGTCCCGTTTTATATGAAACCTTTTTTATTTTAGCCCGTATTAAAAGTAAATTCAGAGA |
| P_yqeZ_ | TTTATACATAAAAAAATGAAACCTTTGATACATTTGTTACGTATGAAGAGAAGGCACTTA |
| P_ythP_ | AATATTATTATGGTTAAAGAAACTTTTTTTATTCTATTTCGTAGTAAATTTTGGAGGTGA |
| P_yuaF_ | TTTGTCTTAAAATTTTGAAACTTTTCCCGAGGTGTCTCGTATAAATGGTAACGGCAGCCG |

Note: The -35 box and -10 box of each promoter were underlined by single underline and double underline, respectively. The transcription start site (TSS) was in red.

Table S4 Spacer sequences between corresponding core region of promoters

| Promoter | Sequences (5’-3’) |
| --- | --- |
| AH(D15) | P_rpoB_-ATTTTTTCAAAAAAT-P_spoVG_ |
| AH(D30) | P_rpoB_- GTGTAACTATATCCTATTTTTTCAAAAAAT-P_spoVG_ |
| AH(D45) | P_rpoB_-CTTTATGACCTAATTGTGTAACTATATCCTATTTTTTCAAAAAAT-P_spoVG_ |
| AH(D60) | P_rpoB_-TGCCCGAAATGAAAGCTTTATGACCTAATTGTGTAACTATATCCTATTTTTTCAAAAAAT-P_spoVG_ |
| AH(D75) | P_rpoB-_TTTTTATTTACCTTATGCCCGAAATGAAAGCTTTATGACCTAATTGTGTAACTATATCCTATTTTTTCAAAAAAT-P_spoVG_ |
| AH(D90) | P_rpoB_-AGAGCCGGGATCACTTTTTTATTTACCTTATGCCCGAAATGAAAGCTTTATGACCTAATTGTGTAACTATATCCTATTTTTTCAAAAAAT-P_spoVG_ |
| WAH(D15) | P_sigW_-P_rpoB_-ATTTTTTCAAAAAAT-P_spoVG_ |
| WAH(D30) | P_sigW_-P_rpoB_- GTGTAACTATATCCTATTTTTTCAAAAAAT-P_spoVG_ |
| WAH(D45) | P_sigW_-P_rpoB_-CTTTATGACCTAATTGTGTAACTATATCCTATTTTTTCAAAAAAT-P_spoVG_ |
| WAH(D60) | P_sigW_-P_rpoB_-TGCCCGAAATGAAAGCTTTATGACCTAATTGTGTAACTATATCCTATTTTTTCAAAAAAT-P_spoVG_ |
| WAH(D75) | P_sigW_-P_rpoB_-TTTTTATTTACCTTATGCCCGAAATGAAAGCTTTATGACCTAATTGTGTAACTATATCCTATTTTTTCAAAAAAT-P_spoVG_ |
| WAH(D90) | P_sigW_-P_rpoB_- AGAGCCGGGATCACTTTTTTATTTACCTTATGCCCGAAATGAAAGCTTTATGACCTAATTGTGTAACTATATCCTATTTTTTCAAAAAAT-P_spoVG_ |
| WAH(U15) | P_sigW_-GCTATTATATCATAA-P_rpoB_-P_spoVG_ |
| WAH(U30) | P_sigW_-TTCAAAAAAAGAAAGGCTATTATATCATAA-P_rpoB_-P_spoVG_ |
| WAH(U45) | P_sigW_-ATGAAGTTTCCGTCGTTCAAAAAAAGAAAGGCTATTATATCATAA-P_rpoB_-P_spoVG_ |
| WAH(U60) | P_sigW_-TCGAGGAACTGTTCGATGAAGTTTCCGTCGTTCAAAAAAAGAAAGGCTATTATATCATAA-P_rpoB_-P_spoVG_ |
| WAH(U75) | P_sigW_-CTGCCATTGAAAAGCTCGAGGAACTGTTCGATGAAGTTTCCGTCGTTCAAAAAAAGAAAGGCTATTATATCATAA-P_rpoB_-P_spoVG_ |
| WAH(U90) | P_sigW_-AGCAAGGCGCGCCTTCTGCCATTGAAAAGCTCGAGGAACTGTTCGATGAAGTTTCCGTCGTTCAAAAAAAGAAAGGCTATTATATCATAA-P_rpoB_-P_spoVG_ |
| AWH(D15) | P_rpoB_-P_sigW_-ATTTTTTCAAAAAAT-P_spoVG_ |
| AWH(D30) | P_rpoB_-P_sigW_-GTGTAACTATATCCTATTTTTTCAAAAAAT-P_spoVG_ |
| AWH(D45) | P_rpoB_-P_sigW_-CTTTATGACCTAATTGTGTAACTATATCCTATTTTTTCAAAAAAT-P_spoVG_ |
| AWH(D60) | P_rpoB_-P_sigW_-TGCCCGAAATGAAAGCTTTATGACCTAATTGTGTAACTATATCCTATTTTTTCAAAAAAT-P_spoVG_ |
| AWH(D75) | P_rpoB_-P_sigW_-TTTTTATTTACCTTATGCCCGAAATGAAAGCTTTATGACCTAATTGTGTAACTATATCCTATTTTTTCAAAAAAT-P_spoVG_ |
| AWH(D90) | P_rpoB_-P_sigW_-AGAGCCGGGATCACTTTTTTATTTACCTTATGCCCGAAATGAAAGCTTTATGACCTAATTGTGTAACTATATCCTATTTTTTCAAAAAAT-P_spoVG_ |
| AWH(U15) | P_rpoB_-AAATTTGATAAACTT-P_sigW_-P_spoVG_ |
| AWH(U30) | P_rpoB_-TCTACACCCTGCCAAAAATTTGATAAACTT-P_sigW_-P_spoVG_ |
| AWH(U45) | P_rpoB_-ATTCACGAATTACCATCTACACCCTGCCAAAAATTTGATAAACTT-P_sigW_-P_spoVG_ |
| AWH(U60) | P_rpoB_-ATGTTAAGGTAGTTTATTCACGAATTACCATCTACACCCTGCCAAAAATTTGATAAACTT-P_sigW_-P_spoVG_ |
| AWH(U75) | P_rpoB_-AATAATTTTAAAAATATGTTAAGGTAGTTTATTCACGAATTACCATCTACACCCTGCCAAAAATTTGATAAACTT-P_sigW_-P_spoVG_ |
| AWH(U90) | P_rpoB_-CTGTTCAGTCTGTATAATAATTTTAAAAATATGTTAAGGTAGTTTATTCACGAATTACCATCTACACCCTGCCAAAAATTTGATAAACTTP_sigW_-P_spoVG_ |

Table S5 RBS design

| RBS | Sequences (5’-3’) | Predicted translation initiation rate (au) |
| --- | --- | --- |
| RBS103 | TCGAACATCATATTTAAAGTGCCGCTACACCACAT | 992.72 |
| RBS503 | TCGAACATCATATTTAAAGTCGGAGTACTCCACAT | 5674.55 |
| RBS104 | TCGAACATCATATTTAAAGTAGGTTCGTTCCACAT | 8163.86 |
| RBS304 | TCGAACATCATATTTAAAGTAAAGGTTACCCACAT | 30769.97 |
| RBS504 | TCGAACATCATATTTAAAGTACGAGGAGTCCACAT | 56900.31 |
| RBS804 | TCGAACATCATATTTAAAGTAATAGGAGTCCACAT | 77620.54 |
| RBS105 | TCGAACATCATATTTAAAGTCGAGGTAAACCACAT | 109196.16 |
| RBS305 | TCGAACATCATATTTAAAGTAAAGGAGTCCCACAT | 280412.07 |
| RBS505 | TCGAACATCATATTTAAAGTAGAGGAGGGCCACAT | 507917.94 |
| RBS805 | TCGAACATCATATTTAAAGTAAGGAGGTTCCACAT | 818339.89 |
| RBS106 | TCGAACATCATATTTAAAGTACAGGAGGTCCACAT | 1011195.57 |
| RBS156 | TCGAACATCATATTTAAAGTCAAGGAGGTCCACAT | 1489100.65 |
| RBS206 | TCGAACATCATATTTAAAGTAAAGGAGGTCCACAT | 2031355.3 |

Note: The variable sequences for RBS library construction were underlined.

Table S6 Other functional sequences used in this study

| Name | Sequences (5’-3’) | Function |
| --- | --- | --- |
| sfGFP | atgagcaaaggagaagaacttttcactggagttgtcccaattcttgttgaattagatggtgatgttaatgggcacaaattttctgtccgtggagagggtgaaggtgatgctacaaacggaaaactcacccttaaatttatttgcactactggaaaactacctgttccgtggccaacacttgtcactactctgacctatggtgttcaatgcttttcccgttatccggatcacatgaaacggcatgactttttcaagagtgccatgcccgaaggttatgtacaggaacgcactatatctttcaaagatgacgggacctacaagacgcgtgctgaagtcaagtttgaaggtgatacccttgttaatcgtatcgagttaaagggtattgattttaaagaagatggaaacattcttggacacaaactcgagtacaactttaactcacacaatgtatacatcacggcagacaaacaaaagaatggaatcaaagctaacttcaaaattcgccacaacgttgaagatggttccgttcaactagcagaccattatcaacaaaatactccaattggcgatggccctgtccttttaccagacaaccattacctgtcgacacaatctgtcctttcgaaagatcccaacgaaaagcgtgaccacatggtccttcttgagtttgtaactgctgctgggattacacatggcatggatgagctctacaaaTAA | Reporter gene |
| PulWB42 | ATGCTAACGGTTCATCGGACGTTTGAAGCATATTTAGATACAATGGAAACGATTACGATTTTAGTACCGAAATCGTATTGCCAAGGGATAGTGCGCTCATTTACCATCCAAACGCCAAACGGAGAACGACGCGCACTACAAATTACGAAACGCGAAGATTTATGGACGAGCATTAAATATGAGTGCATCCCCGATGTTCCGGTGGAAATCGGAAAAAGTTATTTCATTTATGAAGAACATGGGGCGTTTACCGATTTGCAAATGGGAGCGGTTATTCGCACCGAAGCATTTGATGAGCAGTTTTATTATGACGGTGACCTTGGCATTACGTACAGCAACGATGCCACAACGTTTAAACTTTGGGCACCGACAGCAACAGAGGTAAAACTAAAGCTCATTAACAAAGCAGGAAAAGAAGAACAAATCTCGATGCAGCGCGGAGAAAAAGGGGTATGGTGCGCGACAGTACTCGGTAATTTAGATGGGATATACTATACGTATTTAGCGTGTATTAACCTTGTTTGGCGAGAAGCGGTCGACCCGTATGCTGTAGCGGTATCGGTGAATGGAGAATACGGGGTCGTCGTCGATCTCGCCAAAACGCGAGTGCCCAAACCAGCGCTACCTCCGCTTACTGCCCCAGTCGATGCCATTATTTATGAAATGCACGTTCGTGATTTTACAATTGATCCTCATAGCGGCGTTGTCCATAAAGGAAAGTATCTAGGACTGACTGAATTTCCAACAACAGAACCGTCTGGAACAGTGACGGGGCTTGCTTATTTGAAACAACTTGGGGTTACACACGTAGAGCTGCTTCCGGTGAACGATTTTGCCGGGGTCGATGAACGTGAACCGGAAAAAGAGTACAACTGGGGGTACAATCCGCTTCACTACAACGCGCCGGAAGGAAGTTATGCGACCGATCCTTACGATCCGTATGCGCGTATTCATGAATTGAAACGAGCAATTCGCGCTTTGCAGCAAGAAGGTATTCGTGTCATTCTCGATGTCGTTTATAACCACGTTTACATTCGCGAACAGTCGTCACTAGAAAAACTCGTGCCTGGGTATTATTTTCGCCATGATATTTACGGTATGCCATCGAACGGGACAGGGGTCGGAAATGATCTTGCCCCCGAGCGCAAAATGGTTCGCAAGCTCATTGTCGATTCCGTTCGTTTCTGGCTTACAGAGTATGGCATTGATGGGTTTCGTTTTGATTTAATGGGCATTCTCGATATTGACACGATGAAAGAAGTCGAGGCAGTTGTTCGTGCGCTCCATCCATCCGCTCTATTGCTCGGCGAAGGATGGGATTTGCCGACACCATTGCCGTCGGAAAAAAAAGCAACGATGCAAAATGCCCACCTTCTGCCGACGATTGCCTTTTTTAACGATCGGTTTCGCGATTATGTCAAAGGGAGTACGTTTCATTTAGGGGAACAAGGATTTGTTCTAGGAAACAGCGCACATCGCGAACAAGTGAAACGAGTAATCGAAGGAAGCCATCATTTGTTTTCTCAACCAACGCAAACGGTTAACTACGTCGAATCACACGACAACCATACGCTTTGGGACAAAATGAGCATCGCCAACTATTACGAGCGAGAAACCATTCGTAAAAAACGACAAAAATTAGCGACAGCGATGACCTTATTAGCACAAGGCATTCCATTTTTGCATAGCGGCCAAGAATTTTACCGTACAAAACAAGGAGTAGAAAATAGTTATAACGCTCCAGATGACATTAACCGCATCGATTGGACGAGAAAAAGCATGCACGAACAAGACGTCCGTTACGTGCAAGGATTGATTCGGCTGCGGAAATGGCACGGTGCTTTTCGTTTTCAAACAGTGGAAGAAATAAGAAACCATCTTGTATGGCTTGAACCGATGCCGTCGACAGTGCTCGCTTTTCATCTTTACGACGTATCAGCGTATGGGCCGTGGCGTGATATTATTGTCATTCACCATAATGAAGAAACACGGCTAGCAGTTGCGCTCCCTGATGAAGAAAGATGGTATGTCGTATGTGATGAAACAAGGAGTGGAATCGATCCTCTTTACGCGGCGACAAAAAAAATCGAGCTGCAAGGAATTGGAACAGTCGTGCTTGTGAAAGGACTGACTTAA  The code sequences of PulWB42 were optimized for its expression in *Escherichia coli* | Pullulanase |
| TH1 | CCCTCCTGTACTAGGAGGGTATTTTTTT | Terminator |
| lacO | ggaattgtgagcggataacaattcc | LacI operator |
| lacI | atgaaaccagtaacgttatacgatgtcgcagagtatgccggtgtctcttatcagaccgtttcccgcgtggtgaaccaggccagccacgtttctgcgaaaacgcgggaaaaagtggaagcggcgatggcggagctgaattacattcccaaccgcgtggcacaacaactggcgggcaaacagtcgttgctgattggcgttgccacctccagtctggccctgcacgcgccgtcgcaaattgtcgcggcgattaaatctcgcgccgatcaactgggtgccagcgtggtggtgtcgatggtagaacgaagcggcgtcgaagcctgtaaaacggcggtgcacaatcttctcgcgcaacgcgtcagtgggctgatcattaactatccgctggatgaccaggatgccattgctgtggaagctgcctgcactaatgttccggcgttatttcttgatgtctctgaccagacacccatcaacagtattattttctcccatgaagacggtacgcgactgggcgtggagcatctggtcgcattgggtcaccagcaaatcgcgctgttagcgggcccattaagttctgtctcggcgcgtctgcgtctggctggctggcataaatatctcactcgcaatcaaattcagccgatagcggaacgggaaggcgactggagtgccatgtccggttttcaacaaaccatgcaaatgctgaatgagggcatcgttcccactgcgatgctggttgccaacgatcagatggcgctgggcgcaatgcgcgccattaccgagtccgggctgcgcgttggtgcggatatctcggtagtgggatacgacgataccgaagacagctcatgttatatcccgccgttaaccaccatcaaacaggattttcgcctgctggggcaaaccagcgtggaccgcttgctgcaactctctcagggccaggcggtgaagggcaatcagctgttgcccgtctcactggtgaaaagaaaaaccaccctggcgcccaatacgcaaaccgcctctccccgcgcgttggccgattcattaatgcagctggcacgacaggtttcccgactggaaagcgggcagtga | IPTG response repressor |
| xylO | AGTTAGTTTATTGGATAAACAAACTAACT | XylR operator |
| xylR | ATGGTTATTATTCAAATTGCAGATCAAGCTTTAGTAAAAAAAATGAATCAAAAATTAATATTAGATGAAATTTTGAAGAACTCCCCTGTCTCCAGGGCAACTCTCTCTGAGATTACAGGATTAAACAAGTCTACTGTCTCCTCTCAAGTAAATACACTGCTTGAAAAAGATTTTATTTTTGAAATTGGGGCAGGGCAATCTAGAGGCGGCAGAAGACCTGTAATGCTTGTTTTTAATAAGAATGCAGGCTACTCGATTGGTATTGATATAGGAGTCGACTATCTTAACGGAATTCTAACCGACTTAGAAGGAAATATTATTCTCGAGAAGACTTCTGACTTGTCTAGTTCTTCCGCTAGTGAAGTAAAAGAGATTTTATTTGCACTTATTCATGGTTTTGTAACCCATATGCCTGAGTCCCCTTATGGTCTAGTCGGAATAGGAATTTGTGTTCCAGGCCTTGTAGATCGTCATCAGCAAATTATTTTCATGCCTAACTTAAATTGGAATATCAAAGATTTGCAGTTTTTAATTGAGAGTGAGTTTAATGTTCCGGTTTTTGTTGAAAATGAAGCTAATGCAGGAGCATACGGTGAAAAAGTATTTGGTATGACAAAAAACTATGAAAACATCGTTTACATCAGTATTAATATCGGAATTGGAACTGGACTTGTTATTAACAACGAATTGTATAAAGGTGTTCAGGGTTTTTCTGGGGAAATGGGTCATATGACGATAGATTTTAATGGACCCAAATGCAGCTGTGGAAATCGAGGCTGTTGGGAATTATATGCTTCTGAAAAAGCGTTACTGGCTTCGCTCTCTAAAGAAGAAAAGAATATTTCTCGAAAAGAGATTGTGGAACGCGCAAATAAAAATGATGTAGAAATGTTAAATGCACTTCAAAACTTTGGCTTTTATATCGGAATTGGATTAACCAATATCCTTAATACATTTGATATAGAAGCTGTTATCTTGAGAAATCATATAATTGAATCTCATCCCATTGTTTTAAATACGATTAAAAACGAAGTTTCTTCTAGAGTCCATTCTCATTTAGACAATAAATGTGAACTATTGCCTTCTTCGTTAGGAAAAAATGCACCTGCTTTAGGAGCGGTTTCTATCGTTATTGATTCTTTTTTAAGTGTTACCCCTATAAGTTAG | Xylose response repressor |
| agrD | ATGAATACATTATTTAACTTATTTTTTGATTTTATTACTGGGATTTTAAAAAACATTGGTAACATcgcagcttatagtacttgtgacttcATAATGGATGAAGTTGAAGTACCAAAAGAATTAACACAATTACACGAATAA | |
| P3 | TTAATATTTTAACATAAAAAAATTTACAGTTAAGAATAAAAAACGACTAGTTAAGAAAAATTGGAAAATAAATGCTTTTAGCATGTTTTAATATAACTAGATCACAGAGATGTG | |
| agrB | ATGAATTATTTTGATAATAAAATTGACCAGTTTGCCACGTATCTTCAAAAGAGAAATAACTTAGATCATATTCAATTTTTGCAAGTACGATTAGGGATGCAGGTCTTAGCTAAAAATATAGGTAAATTAATTGTTATGTATACTATTGCCTATATTTTAAACATTTTTCTGTTTACGTTAATTACGAATTTAACATTTTATTTAATAAGAAGACATGCACATGGTGCACATGCACCTTCTTCTTTTTGGTGTTATGTAGAAAGTATTATACTATTTATACTTTTACCTTTAGTAATAGTAAATTTTCATATTAACTTTTTAATTATGATTATTTTAACAGTTATTTCTTTAGGTGTAATCTCAGTATATGCTCCTGCAGCAACTAAAAAGAAGCCCATTCCTGTGCGACTTATTAAACGAAAAAAATATTATGCGATTATTGTTAGTTTAACCCTTTTCATTATCACACTTATCATCAAAGAGCCATTTGCCCAATTCATTCAATTAGGCATCATAATAGAAGCTATTACATTATTACCTATTTTCTTTATTAAGGAGGACTTAAAATGA | |
| agrC | ATGGTCCAAACTAGTATGGAATTGTTAAACAGTTACAACTTTGTTTTGTTCGTATTAACTCAAATGATATTAATGTTTACAATACCAGCTATAATTAGTGGTATTAAGTACAGTAAACTTGATTATTTTTTCATCATAGTAATTTCGACATTATCGTTATTTCTATTTAAAATGTTTGATAGCGCGTCCTTAATCATATTAACTTCATTTATTATTATAATGTATTTTGTCAAAATCAAATGGTATTCTATTTTGTTGATTATGACTTCGCAGATTATTCTATACTGTGCTAACTACATGTATATAGTTATATATGCATATATCACCAAAATTTCTGATAGTATATTTGTAATATTCCCTAGCTTTTTTGTAGTTTATGTGACTATTAGTATACTATTCTCATATATAATAAATAGAGTTCTCAAAAAAATTAGCACACCATATCTAATACTAAACAAAGGATTTTTAATAGTTATTTCGACTATCTTACTGCTTACTTTTTCATTATTTTTCTTTTATTCACAAATAAACTCGGATGAAGCTAAAGTAATAAGGCAGTATTCTTTTATTTTTATTGGTATCACTATATTTTTAAGTATATTAACATTTGTTATTTCTCAATTTCTCCTTAAAGAGATGAAATATAAACGTAATCAAGAAGAAATTGAAACCTATTATGAATATACATTGAAGATTGAAGCTATCAACAACGAAATGCGCAAGTTCCGTCATGATTATGTCAATATCTTAACGACACTTTCAGAATACATTCGAGAAGATGACATGCCTGGCCTACGTGATTATTTCAATAAAAATATTGTACCTATGAAAGACAATTTACAAATGAATGCTATAAAATTAAATGGTATCGAGAATCTTAAAGTACGTGAAATTAAAGGCTTAATTACTGCGAAAATTTTACGTGCACAAGAAATGAATATTCCGATTAGTATCGAAATACCCGATGAAGTAAGTAGCATTAACTTGAATATGATCGATTTAAGTCGCAGTATTGGTATTATTCTTGATAATGCAATTGAGGCATCAACTGAAATTGATGACCCTATCATTCGCGTTGCATTTATTGAAAGTGAAAATTCAGTAACGTTTATTGTTATGAATAAATGCGCTGATGATATACCACGCATTCATGAATTGTTCCAAGAAAGTTTTTCTACTAAAGGTGAAGGTCGTGGTTTAGGTCTATCAACTTTAAAAGAAATTGCTGATAATGCAGACAATGTCTTATTAGATACAATTATCGAAAATGGTTTCTTTATTCAAAAAGTTGAAATTATTAACAACTAG | |
| agrA | ATGAAAATTTTCATTTGCGAAGACGATCCAAAACAAAGAGAAAACATGGTTACCATTATTAAAAATTATATAATGATAGAAGAAAAGCCTATGGAAATTGCCCTCGCAACTGATAATCCTTATGAGGTGCTTGAGCAAGCTAAAAATATGAATGACATAGGCTGTTACTTTTTAGATATTCAACTTTCAACCGATATTAATGGTATCAAATTAGGCAGTGAAATTCGTAAGCATGACCCAGTTGGTAACATTATTTTCGTTACGAGTCACAGTGAACTTACCTATTTAACATTTGTCTACAAAGTTGCAGCGATGGATTTTATTTTTAAAGATGATCCAGCTGAATTAAGAACTCGAATTATAGACTGTTTAGAAACTGCACATACACGCTTACAATTGTTGTCTAAAGATAATAGCGTTGAAACGATTGAATTAAAACGTGGCAGTAATTCAGTGTATGTTCAATATGATGATATTATGTTTTTTGAATCATCAACAAAATCTCACAGACTCATTGCCCATTTAGATAACCGTCAAATTGAATTTTATGGTAATTTAAAAGAACTGAGTCAATTAGATGATCGTTTCTTTAGATGTCATAATAGCTTTGTCGTCAATCGCCATAATATTGAATCTATAGATTCGAAAGAGCGAATTGTCTATTTTAAAAATAAAGAACACTGCTATGCATCGGTGAGAAACGTTAAAAAAATATAA | |

**References**

1. Blom, E. J.; Ridder, A. N. J. A.; Lulko, A. T.; Roerdink, J. B. T. M.; Kuipers, O. P. J. P. O., Time-Resolved Transcriptomics and Bioinformatic Analyses Reveal Intrinsic Stress Responses during Batch Culture of Bacillus subtilis. **2011,** *6* (11), e27160.

2. Guan, C.; Cui, W.; Cheng, J.; Zhou, L.; Guo, J.; Hu, X.; Xiao, G.; Zhou, Z. J. M. C. F., Construction and development of an auto-regulatory gene expression system in Bacillus subtilis. **2015,** *14* (1), 150.

3. Nguyen, H. D.; Phan, T. T. P.; Schumann, W. J. C. M., Expression Vectors for the Rapid Purification of Recombinant Proteins in Bacillus subtilis. **2007,** *55* (2), 89-93.

4. Marchand, N.; Collins, C. H., Peptide-based communication system enables Escherichia coli to Bacillus megaterium interspecies signaling. *Biotechnology and bioengineering* **2013,** *110* (11), 3003-12.

5. Yan, X.; Yu, H. J.; Hong, Q.; Li, S. P., Cre/lox system and PCR-based genome engineering in Bacillus subtilis. *Applied and environmental microbiology* **2008,** *74* (17), 5556-62.
